# A Vulnerable Subtype of Dopaminergic Neurons Drives Early Motor Deficits in Parkinson’s Disease

**DOI:** 10.1101/2024.12.20.629776

**Published:** 2024-12-21

**Authors:** Akira Fushiki, David Ng, Zachary R. Lewis, Archana Yadav, Tatiana Saraiva, Luke A. Hammond, Christoph Wirblich, Bosiljka Tasic, Vilas Menon, Joaquim Alves da Silva, Rui M. Costa

**Affiliations:** 1 Allen Institute, Seattle, WA 98109, USA; 2 Zuckerman Mind Brain Behavior Institute, Columbia University, New York, NY 10027, USA; 3 Center for Translational and Computational Neuroimmunology, Department of Neurology Columbia University Irving Medical Center, New York, NY 10032, USA; 4 Champalimaud Research, Champalimaud Foundation, Lisbon 1400-038, Portugal; 5 Department of Neurology, University Hospital of Würzburg, Würzburg 97080, Germany; 6 Department of Neurology, The Ohio State University, Columbus, OH 43210, USA; 7 Department of Microbiology and Immunology, Sidney Kimmel Medical College, Thomas Jefferson University, Philadelphia, PA 19107, USA; 8 NOVA Medical School, Universidade Nova de Lisboa, Lisbon 1169-056, Portugal; 9 Aligning Science Across Parkinson’s (ASAP) Collaborative Research Network, Chevy Chase, MD, 20815

**Keywords:** Parkinson’s disease, dopamine, cell types, neurodegeneration, vulnerability, tremor, motor symptoms

## Abstract

In Parkinson’s disease, dopaminergic neurons (DANs) in the midbrain gradually degenerate, with ventral substantia nigra pars compacta (SNc) DANs exhibiting greater vulnerability. However, it remains unclear whether specific molecular subtypes of ventral SNc DANs are more susceptible to degeneration in PD, and if they contribute to the early motor symptoms associated with the disease. We identified a subtype of *Sox6*+ DANs, *Anxa1*+, which are selectively lost earlier than other DANs, and with a time course that aligns with the development of motor symptoms in MitoPark mice. We generated a knock-in Cre mouse line for *Anxa1*+ DANs and showed differential anatomical inputs and outputs of this population. Further, we found that the inhibition of transmitter release in *Anxa1*+ neurons led to bradykinesia and tremor. This study uncovers the existence of a selectively vulnerable subtype of DANs that is sufficient to drive early motor symptoms in Parkinson’s disease.

## Introduction

Parkinson’s disease (PD) is a progressive neurological disorder that primarily affects movement. It develops gradually, sometimes starting with a barely noticeable tremor in one hand^1^. While tremors, rigidity and akinesia are well-known signs of PD, the most distinctive symptom is bradykinesia, characterized by the slowing of movements. The main pathological hallmark of PD is the loss of dopaminergic neurons (DANs) in the substantia nigra^2,3^ and the progressive reduction of DANs worsens the motor symptoms over time.

DANs in the midbrain are heterogeneous and they differ in their cellular properties and functions^4,5^. This heterogeneity is evident across several dimensions, including their molecular profiles and connectivity^4,6^. Transcriptomic studies have revealed that populations of midbrain DANs can be differentiated by distinct gene expression profiles^4^, a finding that extends beyond the conventional anatomical division of midbrain DANs into the substantia nigra pars compacta (SNc, A9) and the ventral tegmental area (VTA, A10). Recent findings have discovered that molecularly unique subtypes of DANs innervate different subregions of the striatum, suggesting a basis for the variety of behavioral responses linked to these distinct neural pathways^5,6^. In PD, the degeneration of DANs follows a stereotypical pattern where some populations of neurons are more vulnerable than others^7^. Within the SNc, there is a distinction between the ventral-tier and dorsal-tier DANs, where the ventral-tier neurons tend to degenerate first^7^. Other studies have focused on changes in gene expression associated with PD compared to healthy controls^8^. Transcriptomic analysis revealed significant upregulation alpha-synuclein^9^ and downregulation of parkin^10^, which are critical to dopamine synthesis and degradation pathways, respectively. This distinct expression pattern suggests a disruption in the proteostasis network that could be pivotal in disease progression. Additionally, genes related to mitochondrial function showed decreased expression levels, consistent with the known impact of mitochondrial dysfunction in Parkinson’s pathology^11^. This differential gene expression or susceptibility aligns with the recent discovery of the distinct axonal projection patterns identified in dopaminergic neuron subtypes. These patterns determine specific neural circuits affected during disease progression, providing a mechanistic basis for the diverse behavioral changes observed. For instance, subtypes projecting to motor-related areas may influence movement deficits, while those targeting limbic regions might underlie emotional or cognitive disturbances, illustrating how circuit-specific vulnerability translates into varied clinical manifestations^4–6^. However, it is not completely understood if specific molecularly identifiable subtypes of DANs in the ventral SNc are especially vulnerable in PD, and if the loss of function of these early vulnerable populations is just a biomarker of the disease or contributes to the early motor symptoms associated with PD.

Here, we characterized the progression of behavioral symptoms and dopaminergic degeneration in MitoPark mice, a well-established PD mouse model^12^, which replicates key aspects of the disease through the targeted deletion of the *Tfam* (mitochondrial transcription factor A) gene in dopaminergic neurons using the Cre-LoxP system. We characterized their motor behaviors in an open field with a motion sensor and identified bradykinesia and a tremor gradually emerging as MitoPark mice showed progressive dopaminergic degeneration in the SNc. We then performed single-nucleus RNA-sequencing (snRNA-seq) in mutant and matched littermate controls at different ages to identify transcriptomic changes in vulnerable dopaminergic populations, and correlated the susceptibility of different cell types to degeneration with the emergence of behavioral phenotypes. We identified specific dopaminergic cell types, highly ventrally localized *Sox6*+ neurons, whose loss was timeline-wise correlated with the onset of motor symptoms. Further, we found that a subpopulation of the *Sox6*+ neurons – the *Anxa1*+ subset is more vulnerable than other *Sox6*+ populations. We next created an Anxa1-Cre knock-in mouse to study the circuit architecture and function of this dopaminergic neuron subtype. We characterized the anatomical inputs and outputs and observed a specific pattern of strong projections of *Anxa1*+ neurons to the dorsal striatum, not seen in other DAN populations, and observed that this subtype also receives different inputs than others. Finally, we showed that specific functional impairment of *Anxa1*+ neurons is sufficient to induce slowness of movements and motor tremors, corresponding to the early PD-like symptoms. These results suggest that a specific vulnerable subtype of DANs is responsible for a range of early motor symptoms of PD, potentially highlighting the role of different dopaminergic neurons subtypes in the progression of the disease.

## Results

### Progressive development of PD-like motor deficits in MitoPark mice

The MitoPark mouse (genotype: *Tfam-loxP/loxP*, *+/DAT-cre*) is a transgenic model of Parkinson’s disease (PD) where the mitochondrial transcription factor A (*Tfam*) is removed selectively in dopaminergic neurons (DANs)^12^. Loss of *Tfam* in DANs leads to a progressive degeneration of these neurons and the manifestation of motor symptoms that are PD-like^12,13^.

**Figure1.**
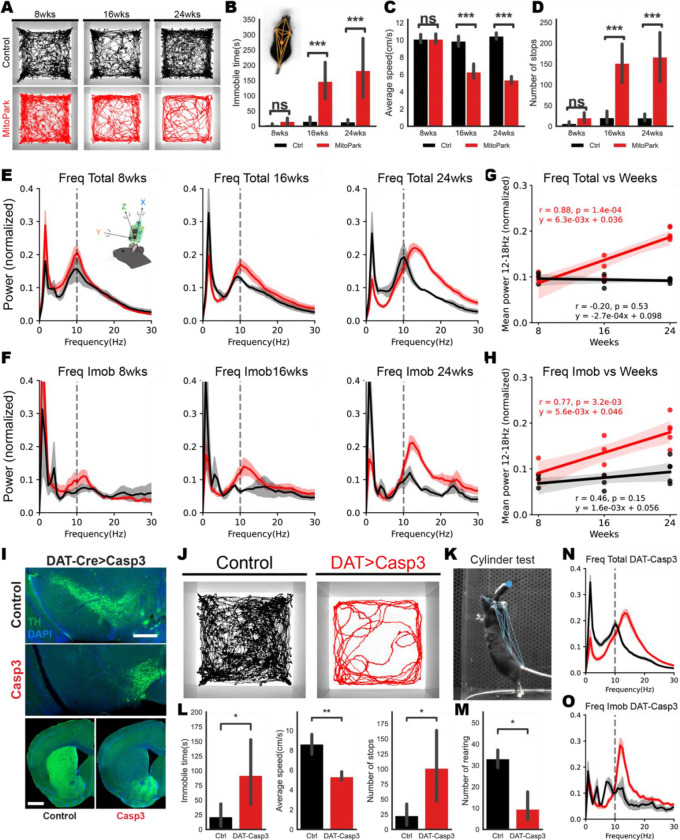
Progressive development of PD-like motor deficits in MitoPark mice **A.** Cumulative trajectories of a representative MitoPark mouse and their control at different stages (8, 16 and 24wks) in the open-field test (black: control animals, red: MitoPark mice). **B-D**. Behavioral characterization of MitoPark mice and their littermate controls at each stage. **(B)** Immobility time: MitoPark mice at later stages spent significantly more time immobile compared to controls. The inset shows tracking performed using DeepLabCut. **(C)** Average speed during mobility: the speed of MitoPark mice was significantly lowered in 16 and 24wks. **(D)** Number of stops: the number of pauses in MitoPark mice showed significant differences compared to the controls. Statistical significance was determined by two-sided Mann–Whitney U-test (***p < 0.001; ns = not statistically significant). Error bars show 95% confidence intervals. MitoPark 8weeks, n=6; Littermate controls 8weeks, n=6; MitoPark 16weeks, n=12; Littermate controls 16weeks, n=8; MitoPark 24weeks, n=9; Littermate controls 24weeks, n=15. **E-H.** Frequency analysis of total time **(E)** or immobility periods **(F)** at different ages of the animals. Mitopark mice showed a pronounced increase in the power of acceleration oscillations at the 12–18Hz range that becomes more prominent in the later stages. **(G, H)** The scatter plots show the correlation between age and mean power in the 12–18Hz frequency band for Mitopark mice and control animals during total period **(G)** or immobile period **(H)**. Pearson’s correlation coefficient reveals a strong positive correlation during both the total period (G: red, r=0.88, p<0.001) and the immobile period (H: red, r=0.77, p<0.01) in MitoPark mice. The solid line represents the linear regression model (G: red, y = 6.3e-03x + 0.036, R² = 0.78; H: red, y = 5.6e-03x + 0.036, R² = 0.60), demonstrating a statistically significant relationship between the two variables in MitoPark mice. MitoPark 8weeks, n=3; Littermate controls 8weeks, n=3; MitoPark 16weeks, n=4; Littermate controls 16weeks, n=3; MitoPark 24weeks, n=5; Littermate controls 24weeks, n=6. **I-O**. Injections of Casp3 virus in the SNc of DAT-Cre mice led to the similar phenotype of MitoPark mice 24 weeks. **(I)** The images demonstrated a specific reduction in dopamine neurons (DANs) within the SNc, as confirmed by staining with anti-TH antibody (green). **(J-M)** The DAT-Casp3 animals showed motor deficits characterized by diminished movement speed, increased incidences of halting and reduced the frequency of rearing behaviors. Statistical significance was determined by two-sided Mann–Whitney U-test (*p < 0.05; ns = not statistically significant). Error bars show 95% confidence intervals. DAT-Casp3 animals, n=5; Controls, n=5. **(N, O)** The virus injection led to the presentation of the same 12–18Hz oscillation seen in MitoPark mice at 24 weeks.

We characterized spontaneous movement of MitoPark mice between 8 and 24 weeks of age in a square open field without any external cues or reward. These mice showed gradual development of locomotor deficits post-natal with decreased average speed (bradykinesia), increased immobility time and increased number of stops (akinesia) (Figures 1A–1D). Given that typical PD has other motor symptoms, such as tremor in addition to bradykinesia and akinesia, we used a wireless motion sensor to detect subtle motor changes that are not easily observable by video-characterization. The sensor measures tri-axial acceleration with high resolution and allows for the continuous monitoring of movement with high frequency (200Hz), which could enable us to detect tremors that are usually poorly documented in genetically engineered rodent models of PD^14^. Given that tremor is defined as an involuntary rhythmic and oscillatory movement of a body part^15^, we explored spectral analysis of accelerometer data to quantify the potential emergence of abnormal oscillations with the progressive degeneration of DANs. Interestingly, we observed a specific oscillation within the 12–18 Hz frequency band that emerged in MitoPark mice at 16 weeks which gradually intensified with age (Figures 1E–1H and S1). Notably, this oscillation was present even when the mice were immobile (Figures 1F and 1H).

We next examined whether depletion of SNc DANs would be sufficient to lead to the development of PD-like symptoms similar to those observed in MitoPark mice. We therefore genetically ablated the neurons in the SNc of DAT-Cre mice by bilateral stereotactic injection of an adeno-associated virus (AAV) to express a Cre-dependent apoptotic gene Caspase-3 (AAV-Ef1a-FLEX-taCasp3)^16^ in the SNc of DAT-Cre mice. Selective losses of the SNc DANs and their projections were observed after 4 weeks of viral expression (Figure 1I). We evaluated mice behavior in the open field, and found that the depletion of SNc DANs reproduced the PD-like phenotype observed in the 24 weeks of MitoPark mice (Figures 1A–1D, 1J and 1L). We also performed a cylinder task to observe rearing behavior, which is more effortful and therefore more likely to be affected in PD, where there is a tendency towards less vigorous movements^17^. We indeed confirmed a significant reduction in rearing in the animals with ablation of SNc DANs (Figures 1K and 1M). Furthermore, we also replicated the emergence of the tremor as observed in MitoPark mice (Figures 1N and 1O). These results suggest that the death of neurons in the SNc is sufficient to produce the bradykinesia, akinesia, and tremor observed in the MitoPark mice.

### Progressive loss of dopamine neurons in the ventral midbrain in MitoPark mice

**Figure 2.**
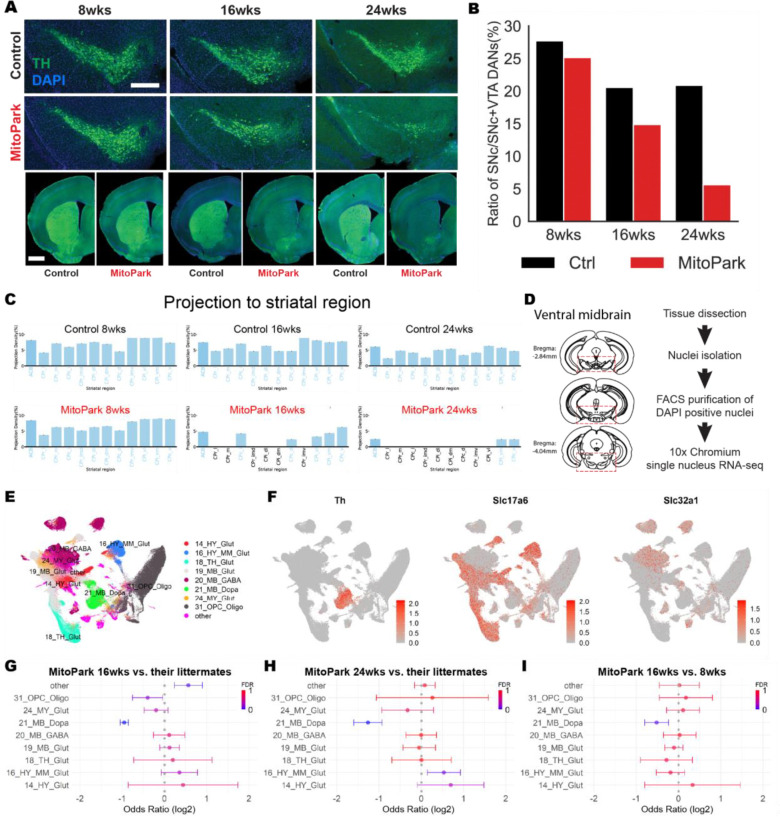
Progressive degeneration of dopamine neurons in MitoPark mice **A-C.** TH immunohistochemistry of MitoPark mice and their littermate controls at 8, 16 and 24 weeks. (A) Histological images of the mice at each stage. Scale bars: 400μm for the substantia nigra (top) and 1mm for the striatum (bottom). (B-C) MitoPark mice exhibited a gradual and preferential loss of DANs in the substantia nigra, and their projections (each n=1). **D.** The workflow for single-nucleus RNA (snRNA) sequencing commenced with the collection of all tissues from the ventral midbrain (red dashed lines). The tissue samples underwent nuclei isolation, followed by the selection of DAPI+ nuclei. These nuclei were subsequently processed using the 10x Genomics platform for sequencing. **E.** UMAP visualization of snRNA-seq data from the ventral midbrain. Data are combined across all animals (MitoPark mice and their controls) and classified by the Allen Brain Cell (ABC) Atlas class annotations. Nuclei that are limited in number or belong to infrequent categories are designated as ‘others.’ **F.** Feature plot showing expression of marker genes used to label the main class of cells: *Th* (DANs), *Slc17a6* (excitatory neurons) and *Slc32a1* (inhibitory neurons). **G-I.** Dots and whiskers represent odds-ratio (OR) with 95% confidence interval (CI) obtained from MASC. OR estimates of major cell types associated with MitoPark mice (color; false discovery rate (FDR)-adjusted P<0.05). **(G)** MitoPark 16 weeks versus their littermate controls; 21_MB_Dopa (OR=−0.95, FDR-adjusted P<0.05). **(H)** MitoPark 24 weeks versus their littermate controls; 21_MB_Dopa (OR=−1.26, FDR-adjusted P<0.05). **(I)** MitoPark 16 weeks versus 8 weeks; 21_MB_Dopa (OR=−0.51, FDR-adjusted P=0.057).

To determine whether the mitochondrial *Tfam* deficit leads to changes in the number of midbrain DANs, we surveyed Th expression in MitoPark mice and monitored changes in *Th*+ cells in the SNc of MitoPark mice at 8, 16 and 24 weeks (Figure 2A and 2B). We also examined Th+ axonal projections from the SNc of MitoPark mice to striatum and performed brain reconstruction. Given that the current Allen Common Coordinate Framework (Allen CCF) does not have anatomical subdivisions of the striatum^18^, we created an updated map based on cortical projections^19^ and applied the imaging analysis pipeline BrainJ as previously described^20^ (Figure S2). This updated atlas allowed us to quantitatively analyze the density of projections neurons in subregions of the striatum and visualize the prominent loss of *Th*+ axonal projections in MitoPark mice (Figure 2C). We observed that the projections from the SNc DANs to the striatum were intact at 8 weeks in MitoPark mice. However, by 16 weeks, their projections were significantly reduced compared to age-matched controls, especially for those targeting the dorsal striatum. By 24 weeks, the projections were largely depleted. (Figure 2A and 2C).

We also employed single-nucleus RNA sequencing (snRNA-seq) of the ventral midbrain of MitoPark mice, their littermate controls (genotype: *Tfam-loxP/loxP*, +/+ or *Tfam-loxP/*+, +/+) and C57BL/6 (B6) mice at 8, 16, and 24 weeks of age (8 mice in each group; 4 males and 4 females). We used a previously described experimental approach to isolate nuclei and profile their transcriptomes from mice^21^ (Figure 2D, see “Methods” section). We obtained a total of 293,435 nuclei, of which 93,703 were from MitoPark mice, 100,266 were from their littermate controls and 99,466 were from B6 mice. We mapped cell annotations to our data with the Allen Brain Cell (ABC) Atlas using their MapMyCells tool^22^ (Figures 2E and S3A). Dopaminergic nuclei were identified as the subclass annotation of 215_SNc_VTA_RAmb_Foxa1_Dopa at the ABC Atlas, within the class annotation of 21_MB_Dopa (Figure S3A). The dopaminergic population was also clearly distinguished by expression of the canonical marker gene, *Th* (Figure 2F). Using *Rbfox3* to mark neuronal cells, we identified other neuronal cell types such as excitatory (*Slc17a6* positive) and inhibitory (*Slc32a1* positive) cell types (Figure 2F and S3B). Oligodendrocytes were characterized by the expression of *Mog*, while Oligodendrocyte precursor cells (OPCs) expressed high levels of *Pdgfra*. Microglia were characterized by *Ptprc* expression, while astrocytes and endothelial cells were marked by specific expression of *Gja1* and *Nostrin*, respectively (Figure S3B). Consistent with our histological findings, we observed a significant decrease in the number of dopaminergic cells in MitoPark mice between 8 weeks and 24 weeks (Figure S4A). To address whether cell types are enriched or depleted in MitoPark mice, we compared the proportions of all cell-types across the ventral midbrain at each stage. We found that the dopaminergic populations significantly decreased with age compared with other cell types (Figure S4B). We also performed mixed-effects modeling (MASC)^23^ that accounts for technical and biological confounders to identify cellular populations associated with the disease models (Figures 2G–2I and S4C–S4E). MitoPak mice had a significant reduction of DANs at 16 and 24 weeks relative to their littermate controls, while the other cell-types were stable (Figures 2G, 2H, S4C and S4D). A similar reduction of DANs was also observed between 8 and 16 weeks (Figure 2I and S4E). Together, these data demonstrate that *Tfam* depletion in MitoPark mice induces robust and selective dopaminergic degeneration without altering the number of other neighboring cell types (Figures 2G–2I and S4B–S4E).

### Vulnerability of *Sox6*+ population during the progression of PD-like phenotypes

**Figure3.**
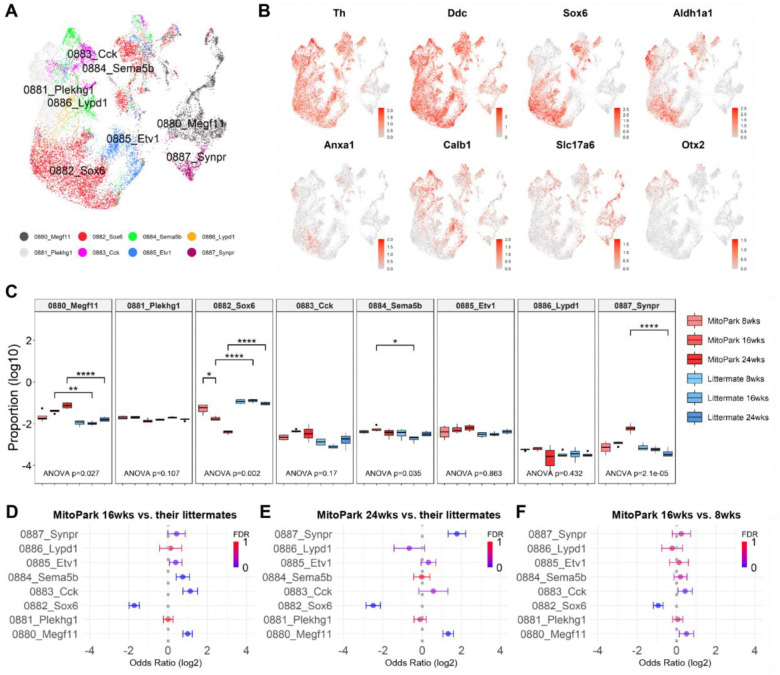
Subtype-specific degeneration within the dopaminergic populations **A.** UMAP representation of 18,611 dopamine neuron nuclei, colored by the supertype annotation of the Allen Brain Cell (ABC) Atlas. In supertype annotation of the ABC Atlas, DANs are largely classified into 8 types. **B.** Representative feature plots showing gene expression associated with dopamine used to label the main class of cells. **C.** Distribution of dopamine subtype proportions across MitoPark and their control animals. 0882_Sox6 is mostly affected among other dopamine neuron subtypes. Statistical significance was determined by two-way ANOVA (p-value indicates the interaction effect between genotype and age) followed by Tukey’s HSD test for multiple comparisons (*p < 0.05, **p < 0.01, ***p < 0.001, ****p < 0.0001). **D-F.** Odds-ratio estimates of each dopamine subpopulation in **(D)** MitoPark 16 weeks versus their littermate controls; 0882_Sox6 (OR=−1.72, FDR-adjusted P<0.05), 0880_Megf11 (OR=1.00, FDR-adjusted P<0.05) and 0883_Cck (OR=1.13, FDR-adjusted P<0.05). **(E)** MitoPark 24 weeks versus their littermate controls; 0882_Sox6 (OR=−2.49, FDR-adjusted P<0.05), 0887_Synpr (OR=1.76, FDR-adjusted P<0.05) and 0880_Megf11 (OR=1.31, FDR-adjusted P<0.05). **(F)** MitoPark 16 weeks versus 8 weeks; 0882_Sox6 (OR=−0.93, FDR-adjusted P=0.057).

We next aimed to identify changes within specific subtypes of DANs during the progression of neurodegeneration. We isolated 18,611 DANs from nuclei of the ventral midbrain, and snRNA-seq data was processed to identify distinct groups representing unique neuronal subtypes (Figures 3A, S5A and S5B). We identified 8 DAN subtypes and confirmed the expression of well-known subtype markers *Sox6*, *Slc17a6, and Calb1* (Figure 3B)^4^. Each group was segregated by distinct genetic markers, which included known dopaminergic neuron subtype-specific genes (Figures S5C, S6A–S6C). To determine which neuronal subtypes were most affected as neurodegeneration progressed, we analyzed the cell composition within each identified group from MitoPark mice and their age-matched controls. Comparative analysis showed significant changes in the composition of dopaminergic neuronal subtypes (Figure 3C). Notably, the proportion of *Sox6*+ neurons was markedly reduced in MitoPark mice, whereas the proportion of other neuron cell types such as *Megf11*+ or *Synpr*+ remained ‘stable’ throughout the degeneration process. As the overall number of dopamine neurons decreases in MitoPark mice, the proportion of these ‘stable’ neurons increased, suggesting they are more resilient compared to other dopamine subtypes that are more susceptible to degeneration (Figure 3C). These neurons are mostly *Vglut2*+ (Slc17a6) (Figures 3A and 3B), which has previously been reported to play a potential neuroprotective role in the survival of DANs in PD model animals^24,25^. Additionally, there are dopamine neuron subtypes that undergo uniform degeneration over time (*Plekhg1*+, *Cck*+, *Sema5b*+, *Etv1*+, *Lypd1*+) (Figures 3C). Using the MASC approach, we further analyzed the differential proportion of different cell-types or states across different groups (Figures 3D–3F) while adjusting for age, sex, and batch as covariates. The *Sox6*+ population showed a significant decrease in the odds ratio in MitoPark mice compared to their littermate controls, especially at 16 or 24 weeks (Figures 3D and 3E), and it was not due to age-related effects alone (Figures S6D–S6G). We also conducted the test across different ages within the same genotype to assess developmental changes in cell population dynamics (Figure 3F). The odds ratio of *Sox6*+ population still declined, suggesting that this subtype exhibits greater vulnerability than other DANs in PD, consistent with findings from human PD transcriptomic studies^8^.

### A subset of *Sox6*+ DANs are more vulnerable in MitoPark mice

**Figure4.**
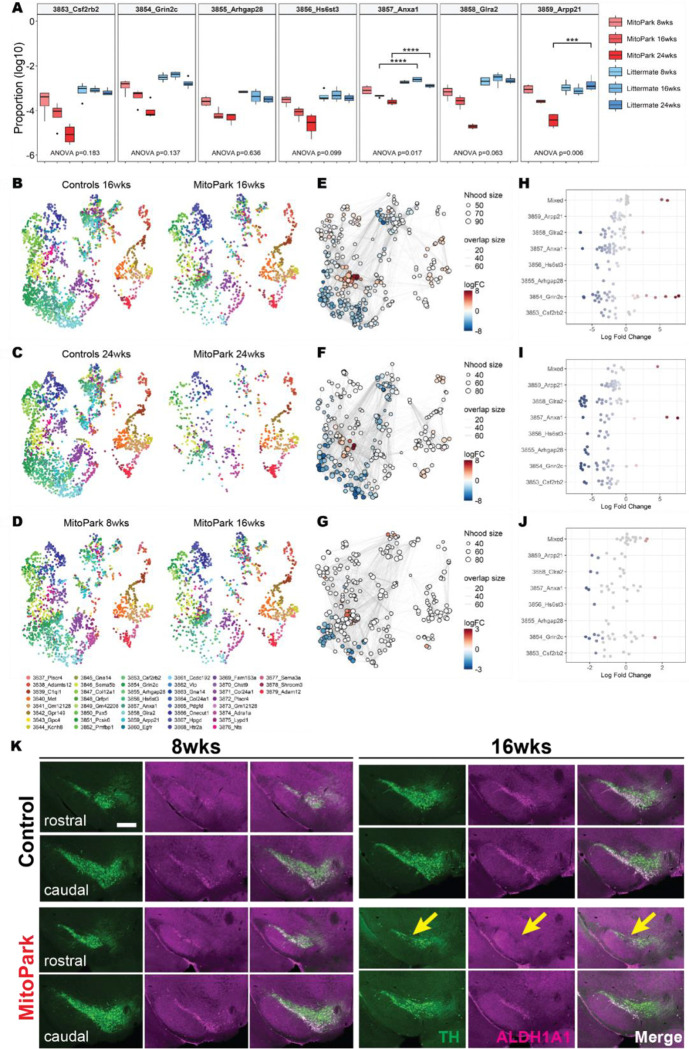
A subset of *Sox6*+ population is more vulnerable than others in MitoPark mice Differential abundance analysis for the dopamine clusters of MitoPark mice and their littermate controls. The analysis reveals a subtype-specific reduction in MitoPark mice at a later stage. **A.** Distribution of *Sox6*+ dopamine subtype proportions across MitoPark and their control animals. 3857_Anxa1 is significantly affected among other dopamine neuron subtypes at 16 and 24 weeks. Statistical significance was determined by two-way ANOVA (p-value indicates the interaction effect between genotype and age) followed by Tukey’s HSD test for multiple comparisons (*p < 0.05, **p < 0.01, ***p < 0.001, ****p < 0.0001). **B-D.** UMAP plots showing the dopamine subpopulations including MitoPark mice and their littermate controls (n=8 at each stage and genotype). Forty-three clusters are annotated by the Allen Brain Cell (ABC) Atlas. **E-G.** Neighborhood (Nhood) graphs with the results from Milo differential abundance testing between MitoPark mice and controls (E:16 weeks, F: 24 weeks), and between MitoPark mice at different stages (G: 8 and 16 weeks). Nodes represent neighborhoods, colored by their log fold change compared to their controls (blue: less abundant, red: more abundant, gray: non-differentially abundant). Graph edges show the number of cells shared between two neighborhoods. **H-J**. Beeswarm plots display the distribution of log-fold changes in cell abundance. Dots representing neighborhoods that overlap with the same cell populations are grouped together. Colors are represented similarly to **E-G**. **K.** Histological images representing 8 and 16 weeks of MitoPark mice reveal that significant cell depletion in the ventral region, particularly in the rostral part of the SNc (TH: green, ALDH1A1: magenta). Scale bar 400μm.

*Sox6* is involved in the development and differentiation of DANs, particularly in the ventral midbrain DANs^26^. The *Sox6*+ supertype (ID: 0882 in 215_SNc_VTA_RAmb_Foxa1_Dopa) can be further subdivided according to their anatomical location, distinct molecular signatures, and functional properties^4^. To explore the diversity of *Sox6*+ neurons, we conducted transcriptomic analysis of the subtypes and performed the cluster annotation based on the ABC atlas^22^. We found 43 clusters (ID: 3837–3879) under the supertype (ID: 0880–0887) annotation, of which, the *Sox6*+ supertype can be divided into 7 subsets (ID: 3853–3859) (Figure S5A). We measured the proportions of the *Sox6*+ dopaminergic subtypes in MitoPark and control animals and found that *Anxa1*+ (ID: 3857) and *Arpp21*+ (ID: 3859) subsets significantly decreased compared to controls, with *Anxa1*+ showing a decrease compared to controls at both 16 and 24 weeks, whereas *Arpp21*+ just showed a significant decrease at 24 weeks (Figure 4A). This suggests that the *Anxa1*+ subtype of *Sox6*+ DANs is especially vulnerable early in the disease. We also performed an additional differential abundance analysis using Milo, a method that offers a structured approach to exploring variations in cell compositions across diverse experimental conditions^27^ (Figures 4B–4J and S7A–S7I). Milo analysis confirmed a significant depletion of *Sox6*+ population in MitoPark mice at 16 and 24 weeks compared to their littermate controls, consistent with previous analyses (Figures S7A–I). We then applied the cluster annotation and found that *Glra2+, Anxa1+, Hs6st3+, Grin2c+ and Csf2rb2*+ subsets exhibited substantial fold changes in gene expression at 16 weeks (Figures 4B, 4E, and 4H). We also performed the analysis between 8 and 16 weeks in MitoPark mice and found these same subpopulations (ie. *Glra2+, Anxa1+, Hs6st3+, Grin2c+ and Csf2rb2*+) were affected (Figures 4D, 4G, 4J, and S7J–S7L). To verify the transcriptional reduction of DAN subsets in the SNc of MitoPark mice, we conducted immunostaining with an antibody targeting *Aldh1a1*, a well-established marker for ventral DANs^4,5^ (Figure 4K). We found a remarkable loss of *Aldh1a1*+ DANs in MitoPark mice at 16 weeks, particularly in the rostral-ventral region (Figure 4K). These results demonstrate that *Sox6*+ DANs exhibited differential susceptibility, with *Anxa1+;Sox6*+ DANs being among the earliest to show vulnerability compared to other *Sox6*+ (ie. *Anxa1−;Sox6*+) DANs.

### Whole-brain efferent and afferent connectivity of *Anxa1*+ DANs

**Figure5.**
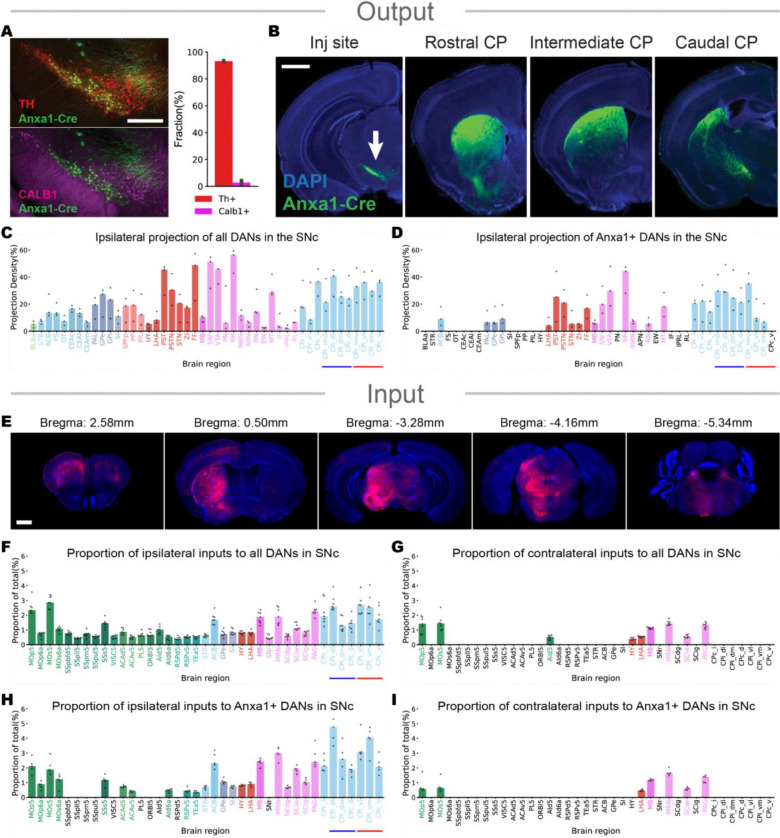
Input-output circuit architecture of *Anxa1*+ DANs in the SNc **A.** In the Anxa1-Cre line, the majority of neurons within the SNc are positive for *Th* and negative for *Calbindin1* (TH, red; CALB1, magenta; n=2). Scale bars: 400μm. **B.** Axonal projections of *Anxa1*+ DANs in the SNc. They preferentially project to dorsal striatum that integrates sensorimotor information from cortical and thalamic regions. Scale bars: 1mm. **C-D.** Brain regions to which all DANs or *Anxa1*+ DANs in the SNc project, measured as the fraction of neurites found within those brain structures defined Allen brain atlas (each n=3). Blue horizontal line indicates dorsal striatum and the red line indicates ventral striatum. **E.** Representative images of rabies infected cells (tdTomato). Rabies virus was injected in the SNc of Anxa1-Cre counterstained with DAPI in blue. Scale bar 1mm. **F-I.** Statistical analysis of the whole-brain distribution of ipsilateral (left) or contralateral (right) monosynaptic inputs to all DANs (F, G) or *Anxa1*+ dopamine neuron subtype (H, I) in the SNc. Average proportion of tdTomato-labeled neurons in approximately 50 brain regions, each with more than 200 cells, was greater than 0.2% of the total input to DANs in DAT-Cre mice (n=7) and Anxa1-Cre mice (n=5; 4 animals for Anxa1-Cre+ and 1 animal for DAT-Flp+;Anxa1-Cre+). Brain areas are color-coded by the Allen Brain Atlas. **[Acronym]** ACAd5, Anterior cingulate area, dorsal part, layer 5; ACAv5, Anterior cingulate area, ventral part, layer 5; ACB, Nucleus accumbens; AId5, Agranular insular area, dorsal part, layer 5; AId6a, Agranular insular area, dorsal part, layer 6a; APN, Anterior pretectal nucleus; BLAa, Basolateral amygdalar nucleus, anterior part; CEAc, Central amygdalar nucleus, capsular part; CEAl, Central amygdalar nucleus, lateral part; CEAm, Central amygdalar nucleus, medial part; CPc_d, Caudal Caudoputamen, dorsal; CPc_i, Caudal Caudoputamen, intermediate; CPc_v, Caudal Caudoputamen, ventral; CPi_dl, Intermediate Caudoputamen, dorsolateral; CPi_dm, Intermediate Caudoputamen, dorsomedial; CPi_vl, Intermediate Caudoputamen, ventrolateral; CPi_vm, Intermediate Caudoputamen, ventromedial; CPr_imd, Rostral Caudoputamen, intermediate dorsal; CPr_imv, Rostral Caudoputamen, intermediate ventral; CPr_l, Rostral Caudoputamen, lateral; CPr_m, Rostral Caudoputamen, medial; EW, EdingerWestphal nucleus; FF, Fields of Forel; FS, Fundus of striatum; Gpe, Globus pallidus, external segment; Gpi, Globus pallidus, internal segment; HY, Hypothalamus; IF, Interfascicular nucleus raphe; IPRL, Interpeduncular nucleus, rostrolateral; LHA, Lateral hypothalamic area; MB, Midbrain; MOp5, Primary motor area, Layer 5; MOp6a, Primary motor area, Layer 6a; MOs5, Secondary motor area, layer 5; MOs6a, Secondary motor area, layer 6a; MRN, Midbrain reticular nucleus; MT, Medial terminal nucleus of the accessory optic tract; ORBl5, Orbital area, lateral part, layer 5; OT, Olfactory tubercle; PAG, Periaqueductal gray; PAL, Pallidum; PIL, Posterior intralaminar thalamic nucleus; PL5, Prelimbic area, layer 5; PN, Paranigral nucleus; PP, Peripeduncular nucleus; PST, Preparasubthalamic nucleus; PSTN, Parasubthalamic nucleus; RL, Rostral linear nucleus raphe; RN, Red nucleus; RR, Midbrain reticular nucleus, retrorubral area; RSPd5, Retrosplenial area, dorsal part, layer 5; RSPv5, Retrosplenial area, ventral part, layer 5; SCdg, Superior colliculus, motor related, deep gray layer; Scig, Superior colliculus, motor related, intermediate gray layer; Sciw, Superior colliculus, motor related, intermediate white layer; SI, Substantia innominata; SNr, Substantia nigra, reticular part; SPFp, Subparafascicular nucleus, parvicellular part; SSpbfd5, Primary somatosensory area, barrel field, layer 5; SSpll5, Primary somatosensory area, lower limb, layer 5; SSpm5, Primary somatosensory area, mouth, layer 5; SSpul5, Primary somatosensory area, upper limb, layer 5; SSs5, Supplemental somatosensory area, layer 5; STN, Subthalamic nucleus; STR, Striatum; TEa5, Temporal association areas, layer 5; VISC5, Visceral area, layer 5; VTA, Ventral tegmental area; ZI, Zona incerta

The results above indicate a subset of *Sox6*+ DANs, the *Anxa1*+ neurons*,* is more vulnerable than others. However, it remains unclear whether loss of these neurons is an early biomarker of disease, or are actually driving early symptoms in PD. To address this, we generated an Anxa1-Cre knock-in mouse line (see ‘Method’ section), with Cre recombinase under transcriptional control of an Anxa1-specific promoter and characterized its expression using GFP to visualize cell bodies and axon projections to output structures. To achieve this, we injected Cre-dependent AAV-GFP into the SNc of Anxa1-Cre mice and counted the number of *Th*+ cells with *Calb1* antibody, which labels the dorsal dopaminergic populations in the SNc (Figures 5A and 5B). In all mice, we observed robust co-localization of GFP with *Th*+ cells but not *Calb1*+ cells, suggesting that most of the GFP+ cells in the SNc are ventral specific DANs (Figures 5A and 5B). We then examined GFP+ axonal projections from the SNc of all DANs or *Anxa1*+ DANs and performed whole brain reconstruction using BrainJ (Figures 5C and 5D). The quantitative analysis revealed prominent axonal projections of *Anxa1*+ DANs to the dorsal striatum (Figure 5D). We also created a DAT-Flp knock-in mouse line (Figure S9A; see ‘Method’ section) and compared the axonal projections of SNc *Anxa1*+ DANs with other DANs subtypes such as *Vglut2*+ or *Calb1*+ (Figure S9B). As shown previously^6^, these subsets have distinct axonal projections to the striatum; *Vglut2*+ DANs project to the dorsolateral and tail striatum, while *Calb1*+ DANs project to the ventral striatum (Figure S9B). We also quantitatively analyzed SNc *Vglut2*+ and *Calb1*+ DAN axonal projections throughout the whole brain, which make specific projections to many different regions. The fact that different populations of DANs have such distinctive projections opens the possibility that different populations modulate different behaviors or behavioral parameters (Figure S9C).

To identify the afferent inputs onto *Anxa1*+ dopaminergic neuronal subtypes and how these inputs differ from other DAN subtypes, we used CVS-N2c rabies tracing, a strain with enhanced transsynaptic labeling^28^, with DAT-Cre+, Anxa1-Cre+ (and also DAT-Flp+;Anxa1-Cre+), DAT-Flp+;Vglut2-Cre+ and DAT-Flp+;Calb1-Cre+ mice. We first examined the inputs to all DANs in the SNc by injecting two Cre-dependent helper viruses (TVA and N2cG) to express the avian receptor (TVA protein) and the rabies glycoprotein G (N2cG) unilaterally into the SNc of DAT-Cre+ mice. Two weeks later, CVS rabies virus (CVS-tdTomato) was injected into the same site (Figure S10A; see “Methods” section). We observed tdTomato-labeled neurons in more brain regions than previous studies^29,30^, especially detected much more inputs for cortical regions, likely attributed to the higher transsynaptic property of the N2C strain. These labeled neurons, primarily restricted ipsilateral to the injection site, represent monosynaptic afferent inputs to the SNc DANs (Figures 5F, 5G and S10C). The same viral injection strategy was also used using Anxa1-Cre+ (and DAT-Flp+;Anxa1-Cre+), DAT-Flp+;Vglut2-Cre+, and DAT-Flp+;Calb1-Cre+ mice, which also labeled an abundant number of tdTomato+ neurons in various parts of brain regions (Figures 5E, 5H, 5I and S9D–9G). The dopaminergic neuron subtypes exhibit differences in their ipsilateral inputs, particularly with respect to inputs from cortical regions. For example, while *Anxa1*+ DANs receive strong inputs from the motor cortex, this subtype does not receive significant inputs from the somatosensory, prelimbic, or orbital cortex. In contrast, *Vglut2*+ DANs receive minimal inputs from the ventral regions of the anterior cingulate and retrosplenial cortices compared to other dopamine subtypes. In most other cases, however, *Anxa1*+ DANs receive similar inputs to the other dopaminergic subtypes, suggesting a large shared organization of inputs into SNc dopaminergic circuits (Figures 5E–5I and S9D–S9G).

### Inhibition of *Anxa1*+ DANs is sufficient to cause early PD-like motor deficits

**Figure6.**
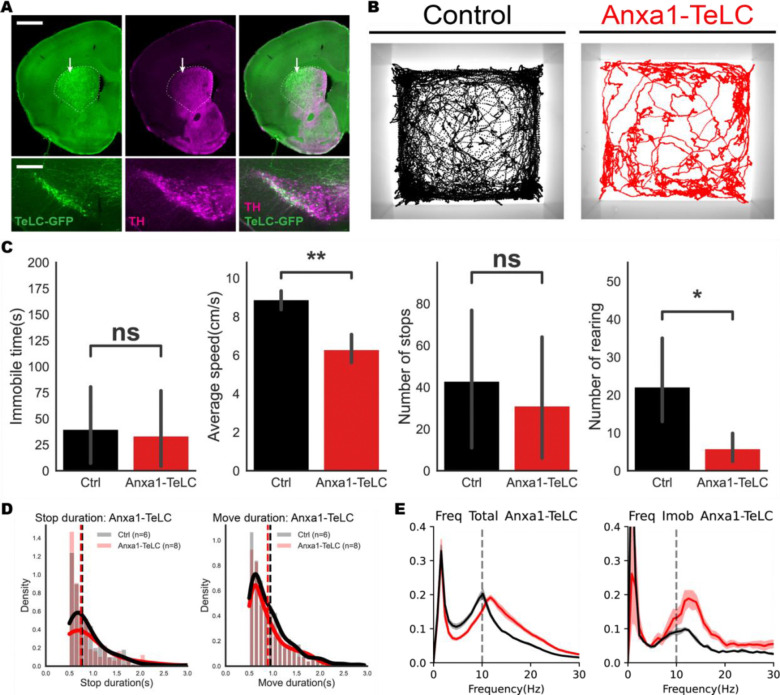
Inhibition of *Anxa1*+ DANs leads to motor deficits resembling early onset PD-like symptoms **A.** In the Anxa1-Cre line, targeted expression of the tetanus toxin light chain (TeLC) specifically eliminated dopamine synaptic transmission in the dorsal striatum (white arrows). Scale bars: 1mm for the striatum (top) and 400μm for the substantia nigra (bottom). **B-D.** Behavioral characterization of Anxa1-TeLC animals. **(B)** Cumulative trajectories of a representative Anxa1-TeLC mouse and their control in the open-field test (black: a control animal, red: Anxa1-TeLC mouse). **(C)** Anxa1-TeLC animals demonstrated slowed movement; however, there were no significant differences observed in terms of immobility duration or the frequency of stops. The frequency of rearing behaviors in the Anxa1-TeLC animals was significantly reduced. Statistical significance was determined by two-sided Mann–Whitney U-test (**p < 0.01, *p < 0.05; ns = not statistically significant). Error bars show 95% confidence intervals. Anxa1-TeLC animals, n=5; Controls, n=4. **(D)** This figure shows the probability density function (PDF) for stop duration (left) and move duration (right), with duration (in seconds) on the x-axis and probability density on the y-axis. There is no significant difference in the density of stop or move durations between Anxa1-TeLC and control animals. **E.** Anxa1-TeLC animals showed the similar 12–18Hz oscillation seen in MitoPark mice at 16 weeks.

To test if the loss of *Anxa1*+ neurons is merely an early biomarker of disease, or could actually be the cause of early symptoms in PD, we selectively silenced synaptic release of *Anxa1*+ DANs with an AAV expressing tetanus toxin^31^ (AAV-FLEX-TeLC-EYFP; Figure 6). We found that silencing of *Anxa1*+ neurons caused a reduction in locomotor speed, suggesting it is sufficient to cause bradykinesia. However, it did not affect immobility time or number of stops, suggesting that it was not sufficient to cause akinesia. (Figures 6B–6D). In a cylinder task, we observed the significant drops in rearing, suggesting that *Anxa1*+ dopaminergic neuron activity is important for effortful movements (Figure 6C). We then performed spectral analysis on mice depleted of *Anxa1*+ SNc dopaminergic neurons and found a slight shift in the frequency of tremor, resembling the phenotype of MitoPark mice at 16 weeks (Figures 6E). Taken together, these data show that inhibition of *Anxa1*+ DANs is sufficient to drive early PD-like motor symptoms in movement speed (bradykinesia), movement vigor, and tremor, but not sufficient to cause akinesia.

## Discussion

The data presented in this study revealed that a subtype of DANs is selectively vulnerable in a mouse model of PD and that its loss is sufficient to drive PD-like motor symptoms that are observed early in the disease. We conducted a precise longitudinal analysis of the behavioral changes exhibited by MitoPark mice, a Parkinson’s disease (PD) model. We identified progressive changes in bradykinesia, akinesia, and in tremor. Concomitantly, these mice demonstrated a progressive degeneration of dopaminergic neurons (DANs) in the midbrain, specifically within the substantia nigra pars compacta (SNc). These phenotypes observed in late-stage MitoPark mice were replicated by ablating dopaminergic neurons in the SNc. Additionally, we performed a comprehensive characterization of the molecular profiles of midbrain neurons as the disease progressed, and uncovered selective vulnerability in the *Sox6*+ DANs, and in particular in the *Anxa1*+ subtype. Finally, we observed that silencing this dopamine subtype was sufficient to produce bradykinesia and tremor of the magnitude observed early in the progression of the degeneration, but not akinesia. This reveals that the loss of these vulnerable neurons is not only a biomarker of early disease, it also drives the early motor symptoms observed early in the disease.

Our behavioral analysis rigorously mapped the progression of motor symptoms in MitoPark mice revealing early symptoms that were not previously reported. Furthermore, it revealed a previous unobserved phenotype associated with dopamine loss. By using a motion sensor in an open field behavioral assay, we have enhanced our capability to detect more subtle motor phenotypes that are not detected using conventional video-based characterization methods. This approach led us to identify tremors in MitoPark mice, which developed incrementally with the progressive degeneration of DANs in the brain. Furthermore, specific ablation and silencing of SNc DANs and the *Anxa1*+ subtype revealed their important role in this tremor phenotype. Although these oscillations were present at rest, the frequency of the tremors we observed are not within the typical band of 4–6 Hz seen in patients with PD. However, tremor frequency differences between species have been well documented, such in the case of the harmaline model of essential tremor with frequencies ranging from 8–10, 10–12 and 11–14 Hz in monkeys, rats and mice respectively^32^. Considering that there are several types of tremors observed in humans and the extent to which these tremors resemble those seen in human diseases has not yet been fully established, a more comprehensive characterization is imperative

We conducted an extensive longitudinal molecular profiling of the cell types in the ventral midbrain as the symptoms progressed in MitoPark mice, utilizing a newly developed atlas, the Allen Brain Cell (ABC) Atlas, for annotation^22^. This detailed examination identified a vulnerable population of DANs, *Sox6*+, the characteristics of which align closely with those identified in previous human studies^8^. In contrast, other subtypes, such as *Megf11*+ (ID: 0880), and *Synpr*+ (ID: 0887) displayed resilience to degeneration. We identified several additional subtypes of *Sox6*+ DANs, with different vulnerability. *Anxa1*+ (ID: 3857) neurons showed the earliest significant decrease compared to WT controls, but *Arpp21*+ (ID: 3859) also showed vulnerability in late stages compared to other subtypes of *Sox6*+ that did not show increased vulnerability. This variance in vulnerability and resilience indicates that the degeneration of DANs occurs at different rates in different types, and that the molecular composition of these subtypes may underly the mechanisms for vulnerability and resilience. Consequently, a deeper understanding of the molecular processes driving the degeneration and resilience of these various DAN subtypes is critical for understanding the progression of the disease, and could be harnessed for the development of biomarkers to monitor progression and eventually the response to disease modifying therapies.

We also characterized with unprecedented depth the afferent and efferent connectivity of three distinct subtypes of DANs in the SNc, roughly representing lateral, dorsal, and ventral SNc DANs, identified by the expression of genes *Vglut2*+, *Calb1*+, and *Anxa1*+ respectively. Consistent with prior findings ^6^, these subtypes exhibit unique projection patterns within the striatum, occupying distinct regions. This supports the conclusion that these subtypes display divergent behaviors in motor control and reward processing^5^. However, our results also reveal that, despite their distinct projection patterns, these subtypes receive surprisingly similar inputs across the brain, with some exceptions of cortical regions. This does not support the idea that different function or vulnerability is related to massively different inputs. One possibility is that despite being subtle, the differences in inputs matter. Another is that positionally, the different populations of DANs receive inputs from different regions of the same area (different neurons, with different functions), and that results in different functions. Finally, it is also possible that neurons in SNc have similar processing functions with subtle differences in their response, but result in different functions because of their more specific output projections. Those projections could target not only differential brain regions, but also different cell types within those regions (e.g. excitatory vs. inhibitory neurons). Another layer of complexity is added by the presence of a recurrent network where SNc DANs receive robust recurrent inhibition from medium spiny neurons from striatum^33,34^ and in turn project to the striatum. This specific projection pattern of each dopaminergic neuron subtype could modulate activity in the striatum, which in turn, through inhibitory feedback, could fine-tune the DAN response during different behaviors or learning processes. Additionally, a more detailed classification of the inputs and projections of single neurons in each DAN subtype, provide the resolution necessary to discern more specific input and output patterns, akin to observations made in fruit flies^35^. This granular approach may reveal intricate neurobiological dynamics obscured when broader classifications are used.

Finally, although targeted manipulation of *Anxa1*+ neurons led to significant bradykinesia and tremor reminiscent of the early stages of disease in MitoPark mice, it did not affect immobile time or akinesia. These discrepancies might be explained by a dosage effect (i.e. akinesia requires the loss of more dopamine neurons than bradykinesia), or by the involvement of other dopaminergic neuron subtypes in different phenotypes. The possibility of homeostatic compensation within DAN neural networks, with different neurons stepping in to sustain the functionality of behaviors affected by the loss of others, should also be discussed. We observed considerable overlap in the brain regions providing afferent connectivity to each dopamine subtype and this observation could be critical to understand the adaptability of these circuits. A critical approach to further unravel these complexities of adaptation as disease develops would involve recording whole-brain activity in Parkinson’s model mice. Gaining a deeper understanding of the resilience and adaptability of neural circuits surrounding dopaminergic neurons will enhance our grasp of Parkinson’s disease and potentially foster innovative therapeutic strategies in the future.

## Materials and Methods

### Contact for reagents and resource sharing

Further information and requests for reagents should be directed to and will be fulfilled by the lead contact, Rui M. Costa (rui.costa@alleninstitute.org).

### Mouse breeding and husbandry

All experimental protocols were performed according to National Institutes of Health (NIH) guidelines and in compliance with the regulations of the Institutional Animal Care and Use Committee at Columbia University. All experimental animals were 2 to 6 month-old mice housed on a 12 hr light/dark cycle with unrestricted access to food and water. Mice used for behavioral experiments were individually housed. The strain and lines used were: C57BL6/J (Jackson Laboratories, 000664), MitoPark mouse^12^, DAT-Cre mouse (Jackson Laboratories, 006660), VGlut2-Cre mouse (Jackson Laboratories, 028863), and Calb1-IRES2-Cre mouse (Jackson Laboratories, 028532). Anxa1-Cre and DAT-Flp mouse lines were developed and utilized in our lab (details are provided below). MitoPark mice (*DAT-Cre*^+/*−*^*;Tfam*^*loxP/loxP*^) were obtained by crossing *DAT-Cre*^+/*−*^*; Tfam*^*loxP/*+^ mice with *Tfam*^*loxP/loxP*^ mice to selectively knockout *Tfam* in dopaminergic neurons.

### Generation of transgenic mice (Anxa1-Cre and DAT-Flp)

Anxa1-Cre and DAT-Flp knock-in mice were generated by Cyagen (Santa Clara, CA) using CRISPR/Cas9. To generate Anxa1-Cre mice, one cell mouse embryos were microinjected with Cas9 protein, a gRNA (5’ GACATCCCAACTATTCTGCA-AGG 3’) targeting exon 13 in the vicinity of the stop codon, and a repair template comprised of homology arms and a ‘P2A-Cre-rBG pA’ cassette. Homology arms were generated by PCR from BAC clones RP23–53N8 and RP23–149B23 as template. Similarly, to generate DAT-Flp mice, a gRNA (5’ CCAACAGCCAATGGCGCAGC-TGG 3’) targeting exon 15, and a template with a ‘2A-FlpO-rBG pA’ cassette were used. Homology arms were PCR amplified from BAC clones RP23–150M11 and RP23–34F24. Two silent mutations, aa 613L (CTG to TTA), and aa 614R (CGC to AGG) were introduced to prevent re-cleavage by Cas9 after homology-directed repair.

### Nuclear suspension, FACS and Single-nucleus RNAseq

We used procedures as previously described^36,37^. Briefly, MitoPark mice and their littermate controls (P56~62 for 8 weeks, P112~118 for 16 weeks and P168~174 for 24 weeks; 4 female and 4 male at each time point) were anaesthetized with isoflurane and perfused with cold artificial cerebrospinal fluid (ACSF) containing N-methyl-d-glucamine (NMDG; 92 mM), KCl (2.5 mM), NaH2PO4 (1.2 mM), NaHCO3 (30 mM), HEPES (20 mM), D-Glucose (25 mM), Sodium L-ascorbate (5 mM), Sodium pyruvate (3 mM), MgSO4 (10mM), CaCl2 (0.5 mM), bubbled with carbogen gas (95% O2 and 5% CO2) at a pH of 7.2–7.4. The brain was sectioned at 400um using a vibratome (VT1200S, Leica Microsystems) on ice, and the ventral midbrain (SNc and VTA) were microdissected from three consecutive sections (−2.48 to −3.88mm from Bregma; Franklin and Paxinos, 2008). The tissues were transferred to microcentrifuge tubes, flash frozen in dry ice with ethanol, and stored at −80C until later use. For nuclei isolation^21^, frozen tissues were placed into a homogenization buffer that consisted of 10mM Tris pH 8.0, 250mM Sucrose, 25mM KCl, 5mM MgCl2, 0.1% Triton-X 100, 1X Protease inhibitor (Promega G6521), 0.5% RNasin Plus RNase inhibitor (Promega N2615) and 0.1mM DTT (Promega P1171). Tissues were placed into a 2ml dounce homogenizer (Sigma D8938) and homogenized using 10 strokes of the loose dounce pestle followed by 10 strokes of the tight pestle to liberate nuclei. Homogenate was strained through a 30μm cell strainer (Miltenyi Biotech 130–098-458) and centrifuged at 900xg for 10 minutes to pellet nuclei. Nuclei were then resuspended in blocking buffer containing 1X PBS supplemented with 0.8% nuclease-free BSA (Sigma, OmniPur 2905) and 0.5% RNasin Plus RNase inhibitor. Prior to fluorescence-activated nuclei sorting (FACS), DAPI (Sigma, D9542) was applied to nuclei suspensions at a final concentration of 0.1μg/ml and nuclei suspensions were filtered through a 30μm nylon mesh (Sysmex, 04–004-2326) to remove aggregates. FACS was performed at the Zuckerman Institute Flow Cytometry platform using a MoFlo Astrios EQ (Beckman Coulter) sorter. Single nuclei were captured by gating DAPI+ events, excluding debris and doublets. Both cell suspension and collection chamber were kept at 4C during sorting. Sorted nuclei were spun down immediately and barcoded using 10x Genomics Chromium Single Cell 3’ Reagent Kit v3 according to the manufacturer’s protocol. Samples were processed and libraries were prepared/sequenced by the Columbia JP Sulzberger Genome Center Single Cell Analysis Core.

### RNAseq data processing and analysis

Sequencing data were aligned and quantified using the Cell Ranger Single-Cell Software Suite (v.6.1.2) with default parameters against the ENSEMBL GRCh38 reference genome^38^. The pre-filtered datasets were then imported into the R Seurat package for downstream analysis (v5.0.2)^39^. We first removed low-quality cells (cell containing < 200 genes and genes expressed in <3 cells of the data). After the filtering, contaminating ambient RNA reads were filtered by using SoupX^40^. Doublets were predicted using the DoubletFinder R package^41^, and were excluded for downstream analysis. After quality control and cell filtering (1000<nFeature_RNA<7500; nCount_RNA<40000; percent_mt<1, percent_ribo<1, percent_hemo<1), the datasets were processed for identifying cell populations by Seurat pipeline. Briefly, pre-processed gene expression matrices were normalized by the NormalizedData function. A total of 2,000 most variable features were then selected with the FindVariableFeatures and scaled by ScaleData command (while regressing out mitochondrial genes) for the Principal Component Analysis (PCA), which was performed by the RunPCA command for 30 PCs. After integration with Harmony^42^, the data was subsequently processed with Louvain clustering and dimensionality reduction via Uniform Manifold Approximation and Projection (UMAP) using 30 principal components, reduction = “harmony”, and resolution of 0.8, to visualize nuclear transcriptomic profiles in two-dimensional space. For annotation, we used the Allen Brain Cell (ABC) Atlas tool MapMyCells (RRID:SCR_024672, the reference taxonomy: 10x Whole Mouse Brain (CCN20230722), mapping algorithm: hierarchical mapping)^22^ to assign neuronal classes or subclasses to nuclei from a whole dataset. To subset the dopaminergic population, we first used the annotation (21_MB_Dopa) to identify and extract the relevant population. We then re-scaled the extracted data and applied the supertype or cluster annotation to the subset and performed further analysis. Differentially expressed genes in each cluster were identified by Wilcoxon test (the FindAllMarkers function in Seurat),

### Differential cell abundance

To identify differentially abundant cell populations in each dataset, we used two methods: MASC (version 0.10)^23^ and Milo (version 1.99)^27^. MASC is a single cell association method for testing whether a certain status influences the membership of single cells while accounting for technical confounds and biological variation. For all datasets, we included sex as a fixed effect, and individual as a random effect in the model. We then tested the significance of association between the status (i.e. MitoPark mice and their littermate controls at each stage, or MitoPark mice at different stages) with the clusters. Cell subpopulations were considered significantly associated with a status at FDR-adjusted P<0.05 and absolute odds ratio >0. Milo is a tool for the test on k-nearest neighbor (KNN) graph neighborhoods. Briefly, we performed PCA dimensionality reduction and KNN graph embedding on the samples. We define a neighborhood as the group of cells that are connected to a sampled cell by an edge in the KNN graph. For each analysis, a k-nearest neighbors (KNN) graph was constructed using the graph slot from the adjacency matrix of previously processed Seurat objects and cells were assigned to neighborhoods (k=30, d=30). For each neighborhood we then perform hypothesis testing between conditions to identify differentially abundant cell states whilst controlling the FDR across the graph neighborhoods. We tested for differences in abundance between the cells at different stages of MitoPark mice and their littermate controls.

### Gene ontology enrichment analysis

We used the g:Profiler web tool and gprofiler2 R package (version 0.2.3)^43^ to identify significantly enriched gene ontology (GO) terms from upregulated or downregulated gene lists in dopaminergic clusters.

### Stereotaxic viral injections

Before starting the surgery mice were subcutaneously injected with Buprenorphine XR (0.5–1 mg per kg body weight). The mouse head was shaved, cleaned with 70% alcohol and iodine, an intradermic injection of bupivacaine (2 mg per kg body weight) was administered, and a small incision from anterior to posterior was made on the skin to allow for aligning the head and drilling the hole for the injection site. Surgeries were performed under sterile conditions and isoflurane (1%–5%, plus oxygen at 1–1.5 l/min) anesthesia on a motorized stereotactic frame (David Kopf Instruments, Model 900SD). Throughout each surgery, mouse body temperature was maintained at 37C using an animal temperature controller (ATC2000, World Precision Instruments). For anterograde tracing experiments, animal were unilaterally injected with 300nL of AAV5-CAG-FLEX-GFP (titer: 4.5E12 vg/mL; UNC Vector Core) into the right hemisphere of the substantia nigra pars compacta (SNc; AP −3.16 mm, ML 1.3 mm, DV −4.0 mm) using a Nanoject III Injector (Drummond Scientific, USA) at a pulse rate of 5.1 nL per second, 20 nL per pulse every 5 s. After injection, the pipette was held in place for 5 minutes before raising to ensure minimize viral efflux. For whole-brain retrograde tracing experiments using rabies virus, 100nL of a 2:1 mixture of the helper viruses, AAV1-CAG-FLEX-N2cG-mKate2.0 (titer: 9.83E12 vg/mL; Janelia Viral Core) or AAVDJ-CAG-FlpX-N2cG-mKate2.0 (titer: 2.60E12 vg/mL; Zuckerman Institute Vector Core) and AAV5-Esyn-DIO-TVA950-EYFP-WPRE (titer: 1.44E13 vg/mL; Salk Viral Vector Core), was injected into the SNc. Two weeks later, 300nl of EnvA-N2c-deltaG-tdTomato (titer: 1.5E9 ffu/ml; Center for Neuroanatomy with Neurotropic Viruses, CNNV) was injected within the same area at a 10-degree angle (SNc; AP −3.16 mm, ML 2.0mm, DV −4.0 mm) to prevent labeling the injection tract with TVA. For monosynaptic labeling from the primary motor cortex or the dorsal striatum, 100nL of a 2:1 mixture of the helper viruses, AAV1-CAG-FLEX-N2cG-mKate2.0 (titer: 9.83E12 vg/mL; Janelia Viral Core) and AAV5-Esyn-DIO-TVA950-EYFP-WPRE (titer: 1.44E13 vg/mL; Salk Viral Vector Core), was injected into the SNc. 100nL of AAVDJ-Ef1a-fDIO-mScarlet (titer: 6.30E12 vg/mL; Zuckerman Institute Vector Core) was injected in the motor cortex or the striatum. Two weeks later, 300nl of EnvA-N2c-deltaG-H2B-HA-FlpO (titer: 1.0E9 ffu/ml; Zuckerman Institute Vector Core) was injected within the same area at a 10-degree angle (SNc; AP −3.16 mm, ML 2.0mm, DV −4.0 mm). After the injection, the skull was cleaned and the skin sealed with sutures and Vetbond tissue adhesive (3M, Maplewood, MN, USA). For ablation experiments, 150 nL of AAV5-EF1a-FLEX-taCasp3-TEVp virus (titer 4.20E12 vg/mL; UNC Vector Core) was bilaterally injected into the SNc of DAT-Cre at two specific coordinates (SNc-medial: AP −3.16 mm, ML 1.10 mm, DV −4.1 mm; SNc-lateral: AP −3.16 mm, ML 1.50 mm, DV −4.1 mm). These coordinates effectively targeted and ablated SNc dopaminergic neurons. For silencing neurotransmission, 300nL of AAVDJ-hSyn-FLEX-TeLC-EYFP (titer 7.40E12 vg/mL; Zuckerman Institute Vector Core) was bilaterally injected in the SNc of Anxa1-Cre (SNc; AP −3.16 mm, ML 1.3 mm, DV −4.0 mm).

### Behavior

Mice were habituated with a wireless motion sensor in a square arena (40 × 40 × 20 cm, length × width × height) housed inside a sound-attenuating chamber for 30 minutes on the first day. Imaging was conducted using a 1280 × 1024 pixel FLIR camera (Point Grey Flea) with a CS mount lens, recording at 30 Hz. For the open-field test, mice were imaged for 30 minutes from a top view, and for the cylinder test (a cylinder arena: 15cm diameter × 30cm length), they were imaged for 15 minutes from a side view. Video acquisition was performed using the commercial software FlyCapture and the open-source visual language Bonsai^44^. Mouse centroid coordinates (i.e., spine2 in DeepLabCut tracking, details provided below) during the open-field recording were used to calculate basic measures of activity (e.g., trajectory or velocity). Acceleration data was collected at a sampling rate of 200 Hz (details provided below).

### Pose estimation and tracking analysis

DeepLabCut^45^ was used to estimate the pose of the mice and track their movements throughout the trials. In the open-field test, seven body parts (nose, right ear, left ear, spine1, spine2, spine3, tail) were labeled to create a skeleton for pose estimation. For the cylinder test, two body parts (motion sensor and nose) were labeled. A total of 1,100 frames were labeled for the open-field test (training: 1.03 million iterations, training error: 1.72 pixels, test error: 2.76 pixels), and 1,040 frames were labeled for the cylinder test (training: 1.03 million iterations, training error: 2.31 pixels, test error: 6.8 pixels). The pose data was filtered using a median filter and verified through visual inspection. For the analysis in the open-field test, to ensure consistent data, we only used data from the 5 to 20-minute mark (15 minutes total) within a 30-minute recording, as mice tend to move differently during the first few minutes after being placed from their home cage into the field. Immobility was defined as any tracking point with a speed of less than 0.5 cm/s for a duration of more than 0.5 seconds, and estimates with a likelihood lower than 0.95 were excluded. Average speed was calculated only during periods when the animal was mobile. In the cylinder test, the full 15 minutes of data were used for analysis, since mice often exhibit exploratory rearing behaviors during the first few minutes. Rearing behaviors were defined by the height of the motion sensor, visible from 360 degrees, and were considered valid if sustained for at least 0.5 seconds in the same position. The height thresholds were determined using DeepLabCut’s output (i.e., filtered trajectory plots), and the number of rearing instances was recorded.

### Motion sensor acceleration data analysis

Wireless motion sensor data was collected using the WEAR system developed by the Champalimaud Hardware Platform. The sensor is compact and lightweight (~1.8 g), capable of sampling nine-axis motion data from a three-axis accelerometer, gyroscope, and magnetometer at rates up to 200 Hz. This device communicates with computers through a base station designed by the platform, utilizing the Harp system. The base station is accessible via a software GUI (e.g., Harp Wear), allowing for easy adjustment of sensor parameters to best fit experimental needs. Additionally, it is compatible with Bonsai software^44^, enabling synchronization of the WEAR sensor data with other data sources, such as cameras. The data was first filtered using a 5^th^ order, 1Hz low-pass Butterworth filter to separate the gravitational acceleration component of each axis. This component was then subtracted to the original acceleration data to obtain the fast acceleration changes induced by mice’s movements (body acceleration). Total body acceleration was determined by calculating the vector norm of the three body acceleration axis. To assess the emergence of rhythmic, oscillatory movements, we performed a spectral analysis of the body’s dorso-ventral acceleration axis. We used the signal.spectrogram function of the Scipy python library^46^ to calculate a spectrogram with consecutive Fourier transforms, using segments of 256 samples with a 50% overlap. The spectrogram was normalized by transforming the power values of each window to a 0–1 range (maximum= 1 and minimum=0). For each mouse a final measure was obtained by calculating the median normalized power considering all the windows of the spectrogram.

### Histology

Mice were deeply anesthetized with isoflurane and transcardially perfused with PBS, followed by ice-cold 4% paraformaldehyde. Brains were then removed for histological analysis. Coronal sections were cut at 30 μm for immunostaining or 75 μm for 3D reconstruction using a Leica VT1000 vibratome. The tissue was rinsed twice in 1x PBS and then permeabilized in PBS containing 0.4% Triton X-100 (PBST). For quantification of *Th*+ cells in MitoPark mice and Anxa1-Cre mouse line, immunohistochemistry was performed with primary antibodies by incubating the sections with an anti-GFP antibody (Chicken, Aves labs Inc, GFP-1020) and a TH antibody (Mouse, ImmunoStar, 22941) diluted at 1:2000 in 0.4% Triton X-100 PBS (PBST) overnight at 4C. To visualize the dorsal-tier or ventral-tier of the SNc DANs, we also used a Calbindin D28K (Rabbit, Synaptic Systems, 214002) or an Aldh1a1 antibody (Goat, R&D Systems, AF5869). The sections were then incubated with secondary antibodies (Donkey anti-Chicken Alexa Fluor 488, JacksonImmunoResearch, 703–545-155; Donkey anti-Goat Alexa Fluor 568, Invitrogen, A11057; Donkey anti-Mouse Alexa Fluor 647, Invitrogen, A31571) diluted at 1:2000 in 0.4% PBST overnight at 4C. DAPI (Sigma D9542; 1:1000) was used as a counterstain in all experiments.

### Image acquisition, processing, and analysis

Coronal sections (30μm or 75 μm) were serially mounted on slides and sections were imaged using an automated slide scanner (Nikon AZ100 Multizoom microscope) equipped with a 4× 0.4NA Plan Apo objective (Nikon Instruments Inc) and P200 slide loader (Prior Scientific), controlled by NIS-Elements using custom acquisition scripts (Nikon Instruments Inc.). Image processing and analysis using BrainJ proceeded as previously described^20^. Briefly, brain sections were aligned and registered using two-dimensional (2D) rigid-body registration. After background subtraction ilastik was used to detect cell bodies and neuronal processes^47^. Probability images for class (soma and neuronal processes) were generated using the pixel classification approach in ilastik, ensuring improved accuracy for segmentation over fluorescence intensity alone. To map the location of these structures to an annotated brain atlas, serial tissue sections were registered into a single volume before 3D registration to the template brain (Allen Brain Atlas Common Coordinate Framework) using Elastix^48^. Initial measurements and data visualization were performed in ImageJ, and subsequent analyses were performed in Python.

### Statistics and reproducibility

All statistical analyses were conducted using R or Python. Data are presented as mean with 95% confidence intervals (CI) or as the standard error of the mean (SEM), unless otherwise specified. Statistical significance was assessed using a two-sided Mann–Whitney U test, and for multiple comparisons, a two-way ANOVA followed by Tukey’s HSD test was applied (*p < 0.05, **p < 0.01, ***p < 0.001, ****p < 0.0001).

## Supplementary Material

Supplement 1

## Data Availability

Single-cell RNA-sequencing data will be deposited in the Gene Expression Omnibus website (https://www.ncbi.nlm.nih.gov/geo/info/seq.html). All other data that support the findings of this study will be available at Zenodo
