# Supplementary material for: A Vulnerable Subtype of Dopaminergic Neurons Drives Early Motor Deficits in Parkinson’s Disease
Source: bioRxiv. 2024 Dec 21:2024.12.20.629776. Preprint. [Version 1] doi: 10.1101/2024.12.20.629776 (PMC11702755; doi:10.1101/2024.12.20.629776)
Supplement: Supplement 1 [file NIHPP2024.12.20.629776v1-supplement-1.pdf]

bioRxiv preprint doi: https://doi.org/10.1101/2024.12.20.629776; this version posted December 21, 2024. The copyright holder for this preprint (which was not certified by peer review) is the author/funder, who has granted bioRxiv a license to display the preprint in perpetuity. It is made available under aCC-BY 4.0 International license.

Figure S1.

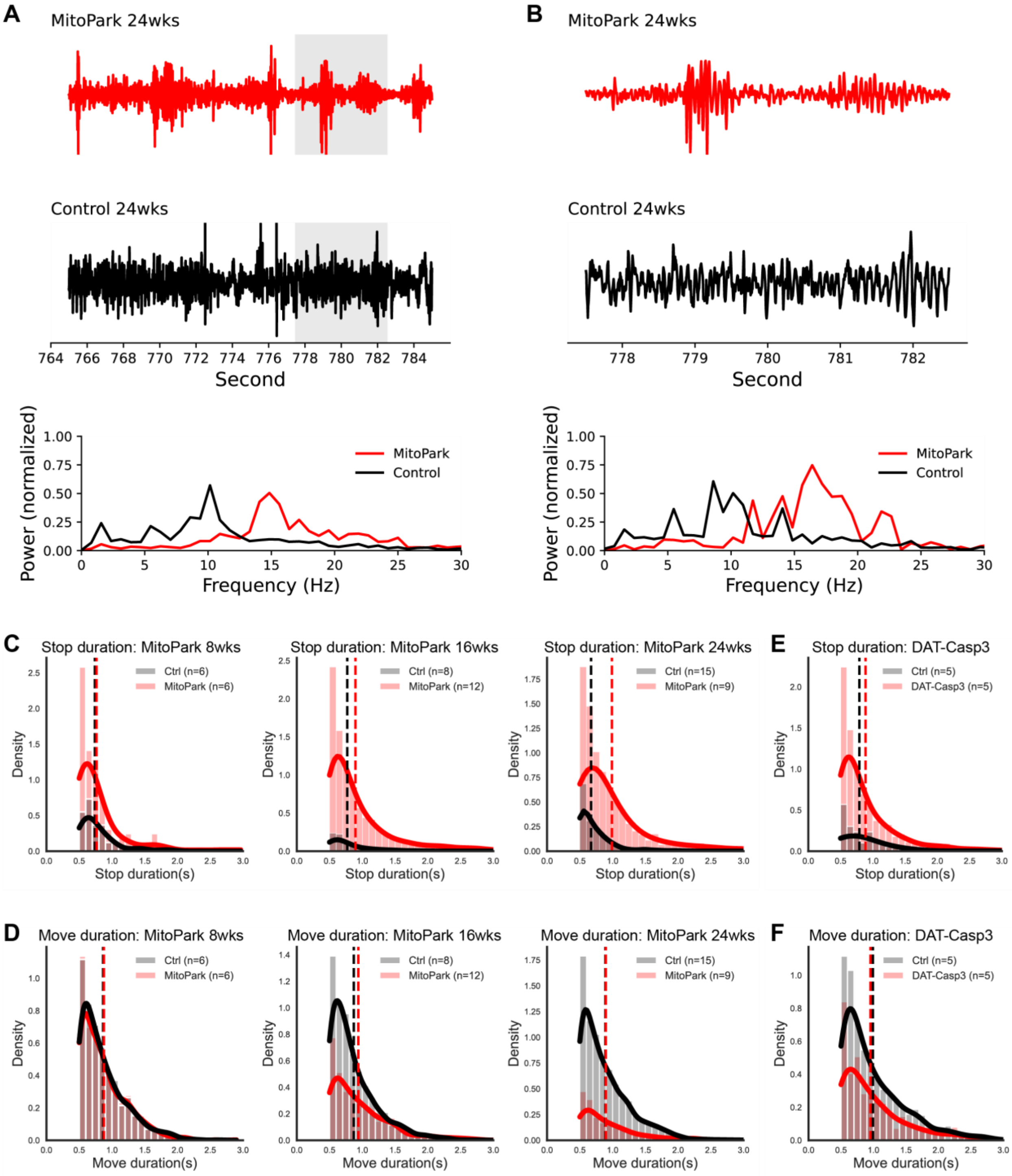

Figure S1. A specific oscillation within the 12-18Hz frequency band in the 24 weeks of MitoPark mice, Related to Figure1.

A-B. Examples of raw traces of dynamic acceleration in MitoPark animal 24 weeks (red) and a control animal (black), with an enlarged view (gray square in A) shown in **B**. MitoPark mice often

bioRxiv preprint doi: https://doi.org/10.1101/2024.12.20.629776; this version posted December 21, 2024. The copyright holder for this preprint (which was not certified by peer review) is the author/funder, who has granted bioRxiv a license to display the preprint in perpetuity. It is made available under aCC-BY 4.0 International license.

show specific 12-18Hz oscillations. **C-D.** These figures show the probability density function (PDF) for stop duration (C) and move duration (D), with duration (in seconds) on the x-axis and probability density on the y-axis. In MitoPark mice, stop duration increased, while move duration decreased at the later stage. **E-F.** The PDF for DAT-Casp3 animals, which also showed an increase in stop duration and a decrease in move duration, similar to the pattern observed in the later stage of MitoPark mice.

bioRxiv preprint doi: https://doi.org/10.1101/2024.12.20.629776; this version posted December 21, 2024. The copyright holder for this preprint (which was not certified by peer review) is the author/funder, who has granted bioRxiv a license to display the preprint in perpetuity. It is made available under aCC-BY 4.0 International license.

Figure S2.

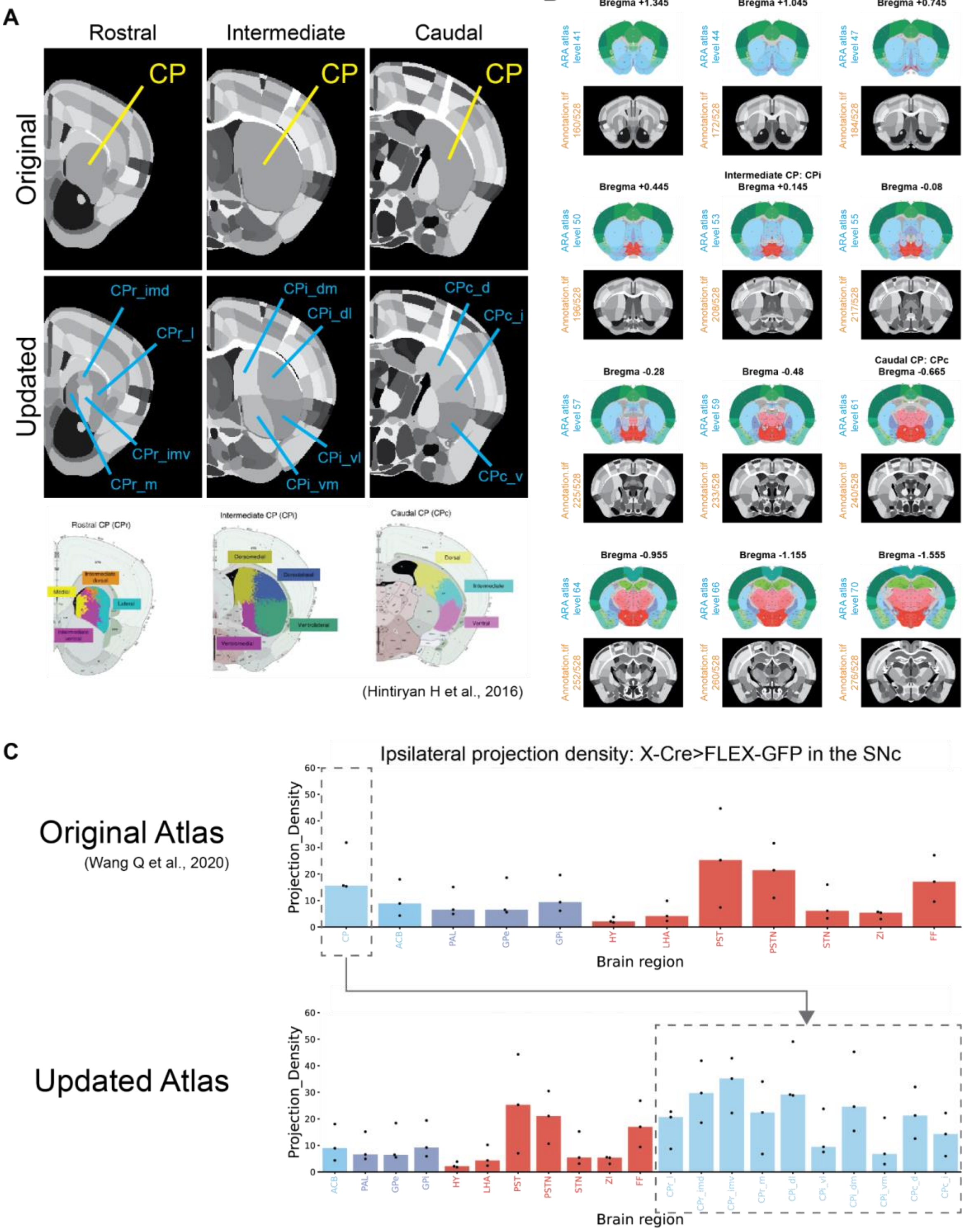

Figure S2. An updated atlas incorporates striatal subregions and enhances the analysis of cells and projections, Related to Figure2 and Figure5.

bioRxiv preprint doi: https://doi.org/10.1101/2024.12.20.629776; this version posted December 21, 2024. The copyright holder for this preprint (which was not certified by peer review) is the author/funder, who has granted bioRxiv a license to display the preprint in perpetuity. It is made available under aCC-BY 4.0 International license.

A. Top: The original Allen Common Coordinate Framework (Allen CCF; version 3 released in 2017). Middle: An updated version of the Allen CCF, incorporating subregions of the striatum (CP: caudate putamen) based on the cortical projections shown at the bottom (CPr_imd: Rostral CP – Intermediate dorsal, CPr_l: Rostral CP – Lateral, CPr_imv: Rostral CP – Intermediate ventral, CPr_m: Rostral CP – Medial, CPi_dm: Intermediate CP – Dorsomedial, CPi_dl: Intermediate CP – Dorsolateral, CPi_vl: Intermediate CP – Ventrolateral, CPi_vm: Intermeidiate CP – Ventromedial, CPc_d: Caudal CP – Dorsal, CPc_i: Caudal CP – Intermediate, CPc_v: Caudal CP – Ventral). **B.** Alignment of coordinates between the ABA (Allen Brain Atlas) and Allen CCFv3, with manually segmented subregions of the striatum. **C**. Comparison of projection density of dopamine neurons in SNc using the original atlas versus the updated atlas. The original atlas (top) does not include subdivisions of the caudate-putamen (CP), while the updated atlas (bottom) defines 11 subregions based on cortical projections, providing a more detailed representation of local projections within the striatum.

bioRxiv preprint doi: https://doi.org/10.1101/2024.12.20.629776; this version posted December 21, 2024. The copyright holder for this preprint (which was not certified by peer review) is the author/funder, who has granted bioRxiv a license to display the preprint in perpetuity. It is made available under aCC-BY 4.0 International license.

Figure S3.

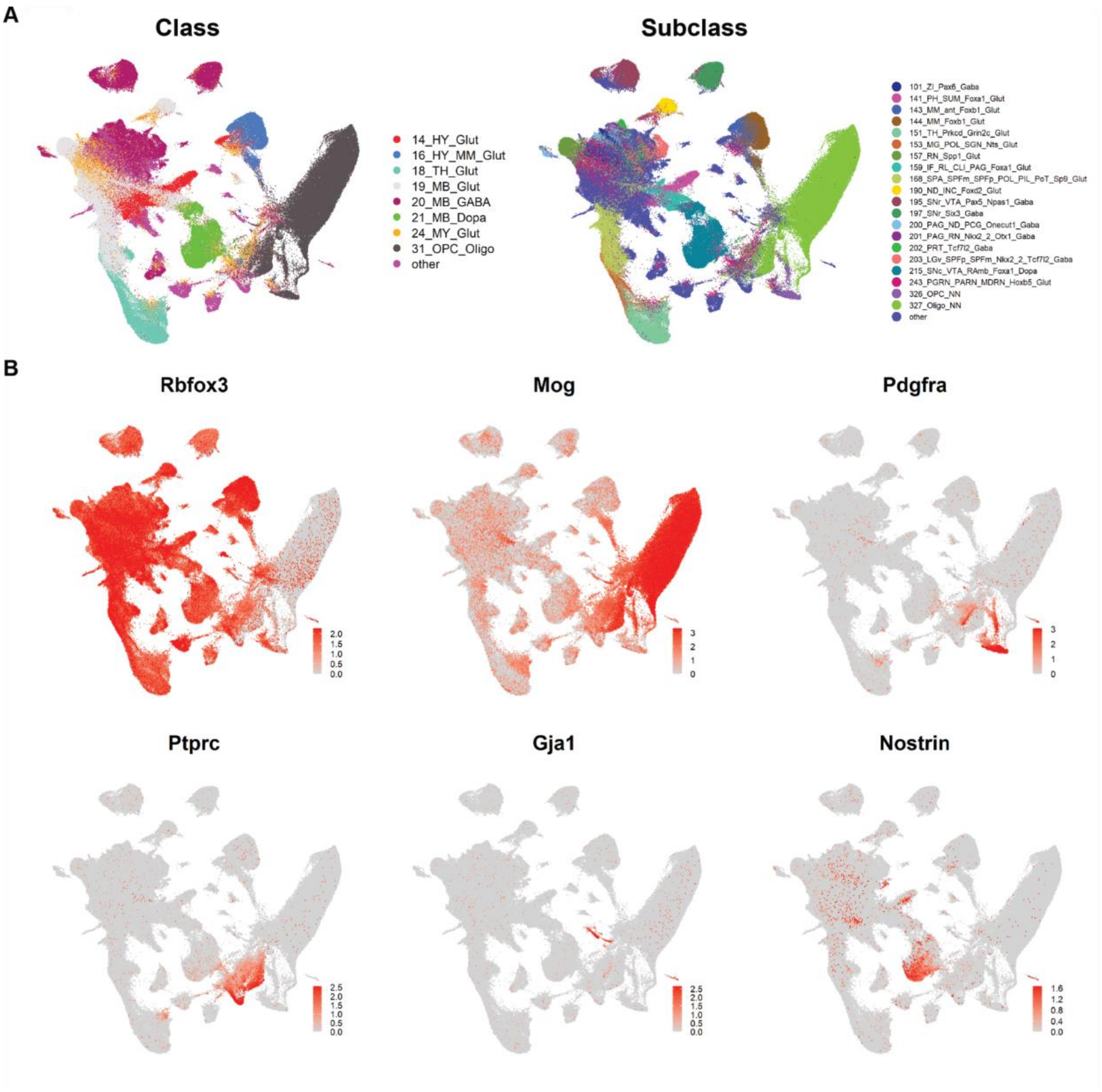

Figure S3. The integrated dataset with the Allen Brain Cell (ABC) Atlas, Related to Figure2.

A. UMAP plots showing all nuclei in the ventral midbrain of MitoPark mice and their controls with the class (left) or subclass (right) annotation of the ABC Atlas. Dopamine cluster is classified as Class: 21_MB_Dopa or Subclass: 215_SNc_VTA_RAmb_Foxa1_Dopa. **B.** Representative feature plots to label main class of cells. Neuronal cells, *Rbfox3*; Oligodendrocytes, *Mog*; Oligodendrocyte precursor cells (OPCs), *Pdgfra*; Microglia, *Ptprc*; Astrocytes, *Gja*; Endothelial cells, *Nostrin*.

bioRxiv preprint doi: https://doi.org/10.1101/2024.12.20.629776; this version posted December 21, 2024. The copyright holder for this preprint (which was not certified by peer review) is the author/funder, who has granted bioRxiv a license to display the preprint in perpetuity. It is made available under aCC-BY 4.0 International license.

Figure S4.

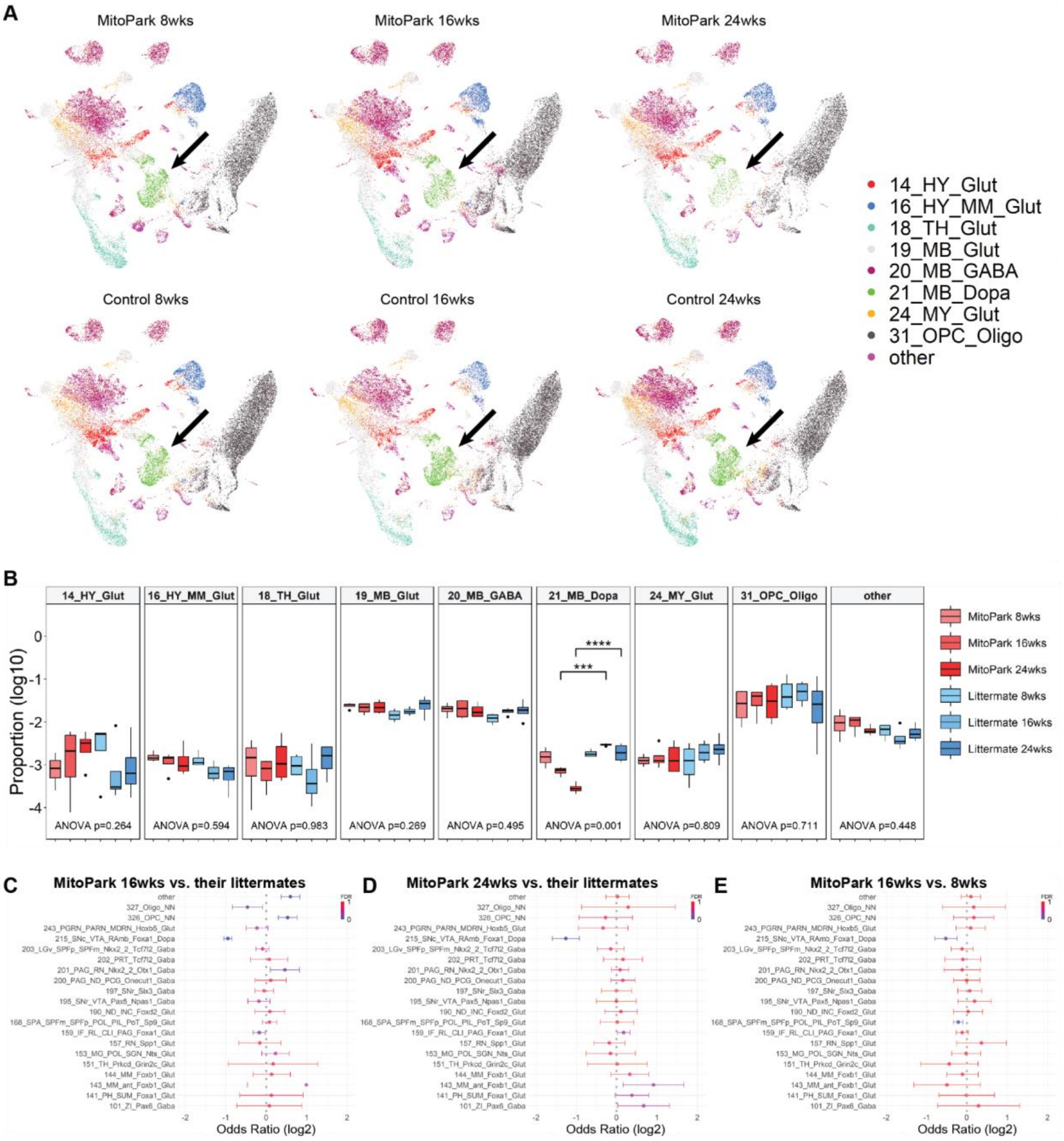

Figure S4. A significant reduction in DANs was observed in the later weeks of MitoPark mice, Related to Figure2.

A. UMAP plots showing all nuclei in the ventral midbrain of MitoPark mice and their controls with the class annotation of the ABC Atlas. Dopamine cluster (black arrow) becomes visibly thinner than other cell types in MitoPark mice. **B.** Box and whisker plots showing the fraction of major cell types originating from the ventral midbrain of MitoPark mice, their littermate controls and B6

bioRxiv preprint doi: https://doi.org/10.1101/2024.12.20.629776; this version posted December 21, 2024. The copyright holder for this preprint (which was not certified by peer review) is the author/funder, who has granted bioRxiv a license to display the preprint in perpetuity. It is made available under aCC-BY 4.0 International license.

WT mice (each n=8), with plot center, box and whiskers corresponding to median, interquartile range (IQR) and 1.5xIQR, respectively. MitoPark mice in the later stages exhibited a significant reduction in the proportion of DANs (21_MB_Dopa), without affecting other cell types. Statistical significance was determined by two-way ANOVA (p-value indicates the interaction effect between genotype and age) followed by Tukey's HSD test for multiple comparisons (*$p < 0.05$, **$p < 0.01$, ***$p < 0.001$, ****$p < 0.0001$). **C-E.** Odds-ratio estimated of major cell types annotated by subclass. **(C)** MitoPark 16 weeks versus their littermate controls; 215_SNc_VTA_RAmb_Foxa1_Dopa (OR=-0.95, FDR-adjusted $P<0.05$). **(D)** MitoPark 24 weeks versus their littermate controls; 215_SNc_VTA_RAmb_Foxa1_Dopa (OR=-1.26, FDR-adjusted $P<0.05$). **(E)** MitoPark 16 weeks versus 8 weeks; 215_SNc_VTA_RAmb_Foxa1_Dopa (OR=-0.51, FDR-adjusted $P=0.086$).

bioRxiv preprint doi: https://doi.org/10.1101/2024.12.20.629776; this version posted December 21, 2024. The copyright holder for this preprint (which was not certified by peer review) is the author/funder, who has granted bioRxiv a license to display the preprint in perpetuity. It is made available under aCC-BY 4.0 International license.

Figure S5.

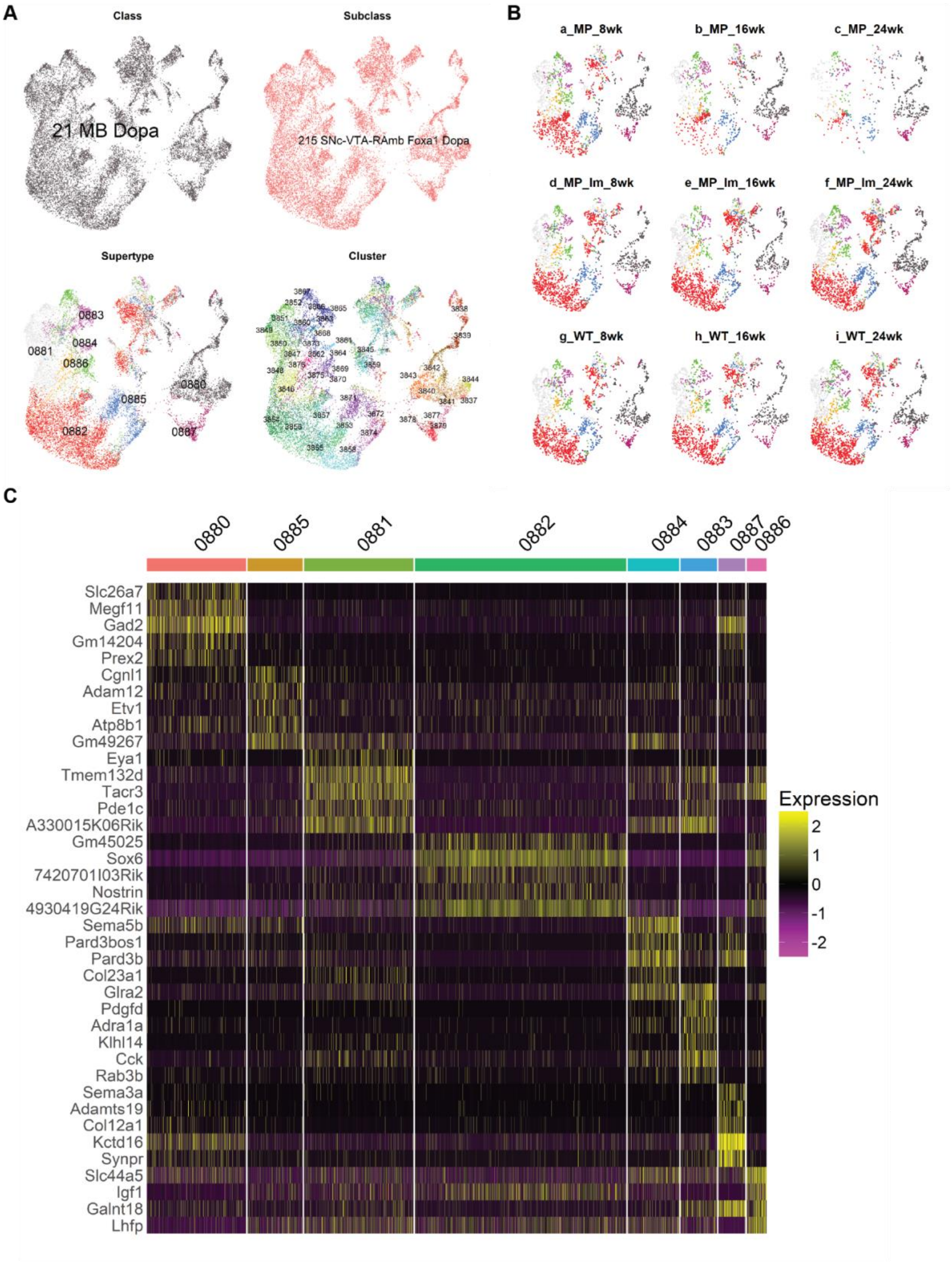

bioRxiv preprint doi: https://doi.org/10.1101/2024.12.20.629776; this version posted December 21, 2024. The copyright holder for this preprint (which was not certified by peer review) is the author/funder, who has granted bioRxiv a license to display the preprint in perpetuity. It is made available under aCC-BY 4.0 International license.

Figure S5. The dopaminergic cluster with distinct marker genes, Related to Figure3.

A. UMAP plots showing dopamine nuclei in the MitoPark mice and their controls with the annotations (Class, Subclass, Supertype and Cluster) of the ABC Atlas. **B.** UMAP plots illustrating the distribution of dopamine nuclei, categorized by supertype annotation based on the species and age. **C.** Top five marker genes for each dopamine neuron subtype categorized by supertype annotation

bioRxiv preprint doi: https://doi.org/10.1101/2024.12.20.629776; this version posted December 21, 2024. The copyright holder for this preprint (which was not certified by peer review) is the author/funder, who has granted bioRxiv a license to display the preprint in perpetuity. It is made available under aCC-BY 4.0 International license.

Figure S6.

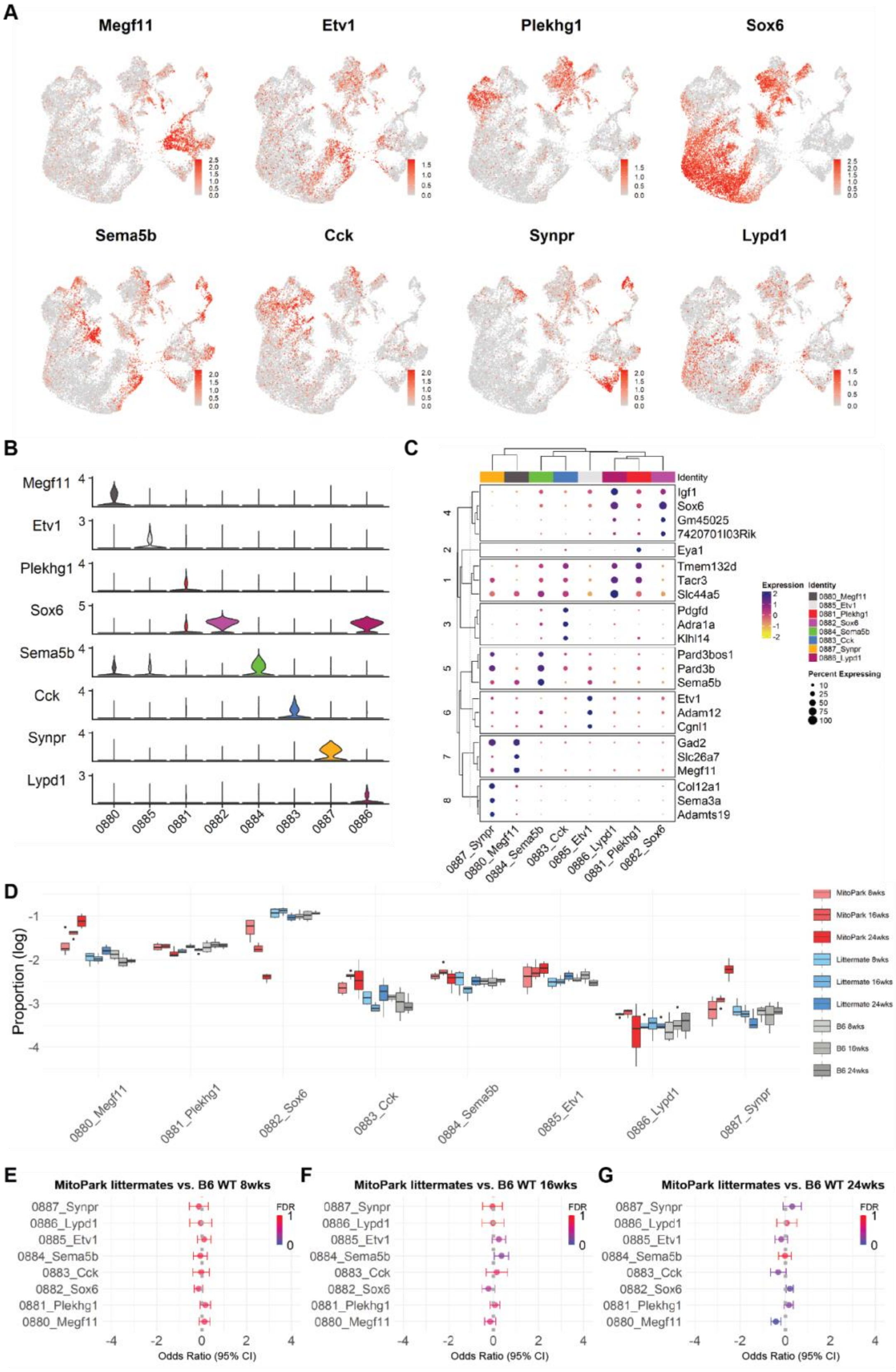

bioRxiv preprint doi: https://doi.org/10.1101/2024.12.20.629776; this version posted December 21, 2024. The copyright holder for this preprint (which was not certified by peer review) is the author/funder, who has granted bioRxiv a license to display the preprint in perpetuity. It is made available under aCC-BY 4.0 International license.

Figure S6. Marker feature expression in the dopaminergic cluster, Related to Figure3.
A. Feature plots depicting the expression of marker genes across dopamine clusters, classified according to supertype annotations.**B.** Violin plot indicating the expression distribution of the top marker gene of each cluster. **C.** Clustered dot plot profiling of marker genes for each dopamine cluster. X-axis represents the dopamine clusters, and the Y-axis shows the top three marker genes. Dot diameter indicates the proportion of cluster nuclei expressing a given gene. The color scale represents the gene expression levels, with black indicating high expression and white indicating low expression. **D.** Distribution of dopamine subtype proportions across MitoPark, their control animals and B6 WT animals. **E-G.** There is no significant age-related degeneration in dopamine populations. **(E)** MitoPark littermate controls versus B6 WT animals at 8 weeks; 0882_Sox6 (OR=-0.14, FDR-adjusted P=0.80). **(E)** MitoPark littermate controls versus B6 WT animals at 16 weeks; 0882_Sox6 (OR=-0.21, FDR-adjusted P<0.41). **(F)** MitoPark littermate controls versus B6 WT animals at 24 weeks; 0882_Sox6 (OR=0.20, FDR-adjusted P<0.11).

bioRxiv preprint doi: https://doi.org/10.1101/2024.12.20.629776; this version posted December 21, 2024. The copyright holder for this preprint (which was not certified by peer review) is the author/funder, who has granted bioRxiv a license to display the preprint in perpetuity. It is made available under aCC-BY 4.0 International license.

Figure S7.

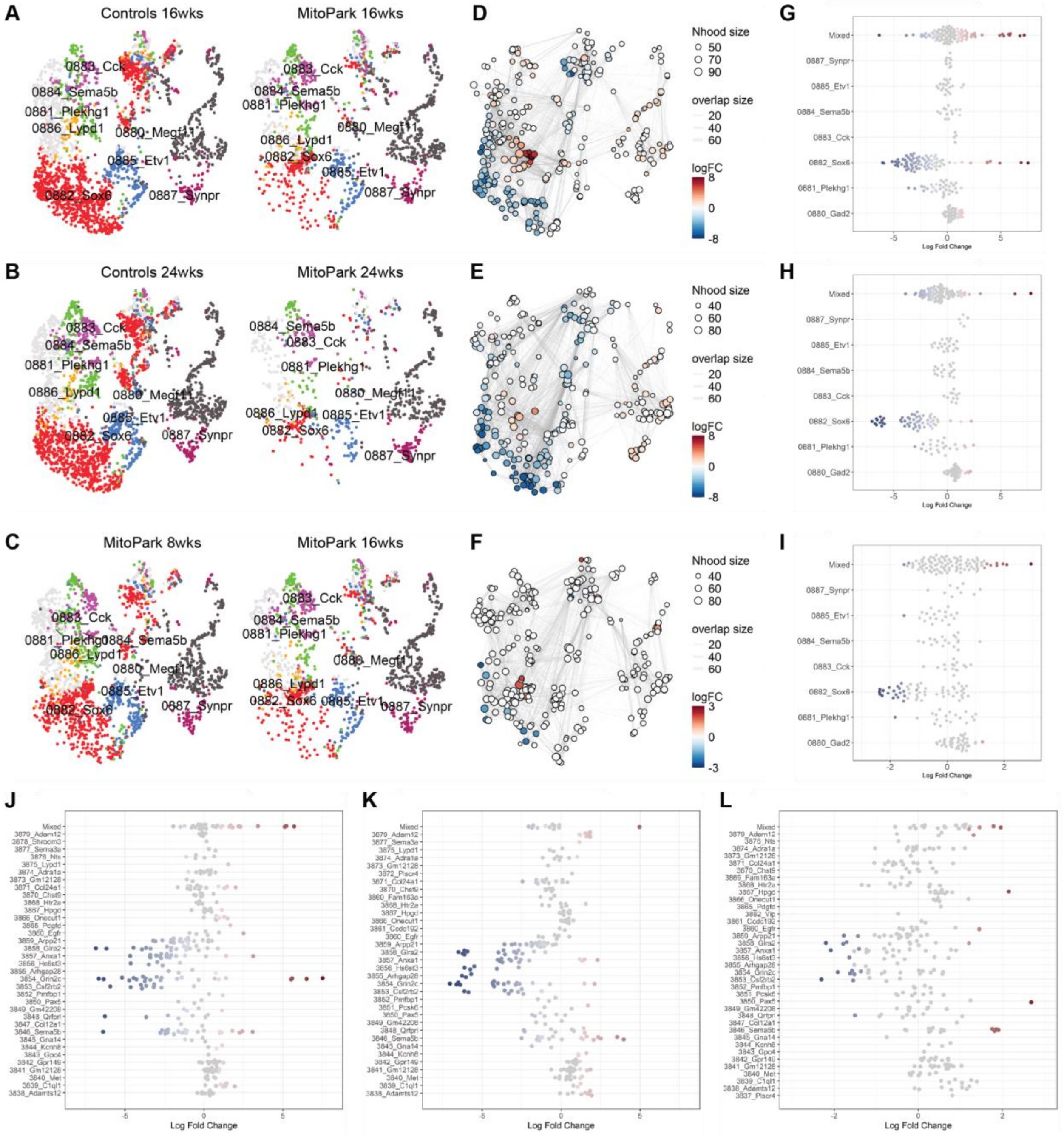

Figure S7. Selective vulnerability of *Sox6*+ dopaminergic population revealed by differential abundance test with Milo, Related to Figure4.

Differential abundance testing for the dopamine subpopulations with supertype annotations by the ABC atlas. **A-C.** UMAP plots showing the dopamine subpopulations of MitoPark mice and their controls. **D-F.** Neighborhood graphs with the results from Milo differential abundance analysis between MitoPark mice and their littermate controls (D:16 weeks, E: 24 weeks), and

bioRxiv preprint doi: https://doi.org/10.1101/2024.12.20.629776; this version posted December 21, 2024. The copyright holder for this preprint (which was not certified by peer review) is the author/funder, who has granted bioRxiv a license to display the preprint in perpetuity. It is made available under aCC-BY 4.0 International license.

between MitoPark mice at different stage (F: 8 weeks and 16 weeks). **G-I**. Beeswarm plots showing the distribution of log-fold changes in each supertype group. Colors are represented similarly to **D-F**. **J-L**. Beeswarm plots showing the distribution of log-fold changes in each cluster. Colors are represented similarly to **Figure4D-F**.

bioRxiv preprint doi: https://doi.org/10.1101/2024.12.20.629776; this version posted December 21, 2024. The copyright holder for this preprint (which was not certified by peer review) is the author/funder, who has granted bioRxiv a license to display the preprint in perpetuity. It is made available under aCC-BY 4.0 International license.

Figure S8.

A

GO Table
Based on dopamine subcluster

Source	Term name	Term ID	Cluster	log10(1/padj)
GO:BP	nervous system development	GO:0007399	3854_Grin2c	85.68
GO:BP	neurogenesis	GO:0022008	3854_Grin2c	76.55
GO:BP	generation of neurons	GO:0048699	3854_Grin2c	74.69
GO:CC	cell junction	GO:0030054	3854_Grin2c	111.69
GO:CC	synapse	GO:0045202	3854_Grin2c	97.04
GO:CC	cytoplasm	GO:0005737	3854_Grin2c	79.54
GO:MF	protein binding	GO:0005515	3854_Grin2c	85.87
GO:MF	binding	GO:0005488	3854_Grin2c	33.21
GO:MF	ion binding	GO:0043167	3854_Grin2c	32.86
HP	Abnormal muscle tone	HP:0003808	3854_Grin2c	5.27
HP	Abnormal nervous system physiology	HP:0012638	3854_Grin2c	5.27
HP	Abnormality of mental function	HP:0011446	3854_Grin2c	4.99
KEGG	Axon guidance	KEGG:04360	3854_Grin2c	9.54
KEGG	Glutamatergic synapse	KEGG:04724	3854_Grin2c	8.91
KEGG	Morphine addiction	KEGG:05032	3854_Grin2c	8.70
MIRNA	mmu-miR-15a-5p	MIRNA:mmu-miR-15a-5p	3854_Grin2c	3.45
MIRNA	mmu-miR-340-5p	MIRNA:mmu-miR-340-5p	3854_Grin2c	3.33
MIRNA	mmu-miR-149-5p	MIRNA:mmu-miR-149-5p	3854_Grin2c	3.01
REAC	Neuronal System	REAC:R-MMU-112316	3854_Grin2c	19.47
REAC	Transmission across Chemical Synapses	REAC:R-MMU-112315	3854_Grin2c	10.89
REAC	Protein-protein interactions at synapses	REAC:R-MMU-6794362	3854_Grin2c	8.66
TF	Factor: ZF5; motif: GSGCGCGR; match class: 1	TF:M00716_1	3854_Grin2c	112.68
TF	Factor: ZF5; motif: GSGCGCGR	TF:M00716	3854_Grin2c	98.98
TF	Factor: ZF5; motif: NRNGNGCGCGCWN; match class: 1	TF:M00333_1	3854_Grin2c	95.72
WP	Dravet syndrome Scn1a A1783V point mutation model	WP:WP5298	3854_Grin2c	4.88
WP	Splicing factor NOVA regulated synaptic proteins	WP:WP1983	3854_Grin2c	3.98
WP	G protein signaling pathways	WP:WP232	3854_Grin2c	3.49

B

GO Table
Based on dopamine subcluster

Source	Term name	Term ID	Cluster	log10(1/padj)
GO:BP	nervous system development	GO:0007399	3855_Arhgap28	72.23
GO:BP	neurogenesis	GO:0022008	3855_Arhgap28	62.71
GO:BP	system development	GO:0048731	3855_Arhgap28	59.98
GO:CC	cell junction	GO:0030054	3855_Arhgap28	62.84
GO:CC	neuron projection	GO:0043005	3855_Arhgap28	59.86
GO:CC	synapse	GO:0045202	3855_Arhgap28	56.83
GO:MF	protein binding	GO:0005515	3855_Arhgap28	39.92
GO:MF	cytoskeletal protein binding	GO:0008092	3855_Arhgap28	19.07
GO:MF	binding	GO:0005488	3855_Arhgap28	18.82
HP	Tremor	HP:0001337	3855_Arhgap28	4.95
HP	Myoclonus	HP:0001336	3855_Arhgap28	3.86
HP	Involuntary movements	HP:0004305	3855_Arhgap28	3.84
KEGG	Morphine addiction	KEGG:05032	3855_Arhgap28	9.82
KEGG	Glutamatergic synapse	KEGG:04724	3855_Arhgap28	7.65
KEGG	GABAergic synapse	KEGG:04727	3855_Arhgap28	7.18
MIRNA	mmu-miR-758-3p	MIRNA:mmu-miR-758-3p	3855_Arhgap28	3.94
MIRNA	mmu-miR-425-5p	MIRNA:mmu-miR-425-5p	3855_Arhgap28	2.87
REAC	Neuronal System	REAC:R-MMU-112316	3855_Arhgap28	15.38
REAC	Transmission across Chemical Synapses	REAC:R-MMU-112315	3855_Arhgap28	9.58
REAC	Neurotransmitter receptors and postsynaptic signal transmission	REAC:R-MMU-112314	3855_Arhgap28	2.62
TF	Factor: Sp1; motif: GGNGGGGGNGGGGGMGGGGCNGGG	TF:M10375	3855_Arhgap28	33.09
TF	Factor: CTCF; motif: NAGGGGGCGCNNKNNNN; match class: 1	TF:M08995_1	3855_Arhgap28	27.03
TF	Factor: ZF5; motif: NRNGNGCGCGCWN; match class: 1	TF:M00333_1	3855_Arhgap28	25.14
WP	Dravet syndrome Scn1a A1783V point mutation model	WP:WP5298	3855_Arhgap28	5.10
WP	Dopaminergic neurogenesis	WP:WP1498	3855_Arhgap28	3.95
WP	Splicing factor NOVA regulated synaptic proteins	WP:WP1983	3855_Arhgap28	3.32

C

GO Table
Based on dopamine subcluster

Source	Term name	Term ID	Cluster	log10(1/padj)
CORUM	Sh3kbp1-Cblb-Egfr complex	CORUM:5172	3857_Anxa1	1.30
GO:BP	nervous system development	GO:0007399	3857_Anxa1	91.15
GO:BP	neurogenesis	GO:0022008	3857_Anxa1	83.86
GO:BP	system development	GO:0048731	3857_Anxa1	82.54
GO:CC	cell junction	GO:0030054	3857_Anxa1	113.87
GO:CC	synapse	GO:0045202	3857_Anxa1	99.92
GO:CC	neuron projection	GO:0043005	3857_Anxa1	89.89
GO:MF	protein binding	GO:0005515	3857_Anxa1	74.58
GO:MF	binding	GO:0005488	3857_Anxa1	32.25
GO:MF	ion binding	GO:0043167	3857_Anxa1	25.68
HP	Autistic behavior	HP:0000729	3857_Anxa1	7.33
HP	Abnormal communication	HP:0034434	3857_Anxa1	5.91
HP	Abnormality of speech or vocalization	HP:0002167	3857_Anxa1	5.58
KEGG	Axon guidance	KEGG:04360	3857_Anxa1	9.78
KEGG	GABAergic synapse	KEGG:04727	3857_Anxa1	9.63
KEGG	Glutamatergic synapse	KEGG:04724	3857_Anxa1	9.39
MIRNA	mmu-miR-758-3p	MIRNA:mmu-miR-758-3p	3857_Anxa1	2.53
MIRNA	mmu-miR-425-5p	MIRNA:mmu-miR-425-5p	3857_Anxa1	2.44
MIRNA	mmu-miR-9-5p	MIRNA:mmu-miR-9-5p	3857_Anxa1	2.41
REAC	Neuronal System	REAC:R-MMU-112316	3857_Anxa1	29.87
REAC	Transmission across Chemical Synapses	REAC:R-MMU-112315	3857_Anxa1	19.52
REAC	Neurotransmitter receptors and postsynaptic signal transmission	REAC:R-MMU-112314	3857_Anxa1	10.51
TF	Factor: ZF5; motif: GSGCGCGR; match class: 1	TF:M00716_1	3857_Anxa1	75.40
TF	Factor: ZF5; motif: GSGCGCGR	TF:M00716	3857_Anxa1	65.82
TF	Factor: ZF5; motif: NRNGNGCGCGCWN; match class: 1	TF:M00333_1	3857_Anxa1	63.72
WP	Dravet syndrome Scn1a A1783V point mutation model	WP:WP5298	3857_Anxa1	8.91
WP	Calcium regulation in cardiac cells	WP:WP553	3857_Anxa1	6.34
WP	Cholesterol biosynthesis	WP:WP103	3857_Anxa1	6.15

D

GO Table
Based on dopamine subcluster

Source	Term name	Term ID	Cluster	log10(1/padj)
CORUM	Caveolar macromolecular signaling complex	CORUM:384	3858_Glra2	1.31
CORUM	Caveolar macromolecular signaling complex, using anti-Cav-3	CORUM:385	3858_Glra2	1.31
GO:BP	system development	GO:0048731	3858_Glra2	32.92
GO:BP	generation of neurons	GO:0048699	3858_Glra2	32.89
GO:BP	neuron differentiation	GO:0030182	3858_Glra2	32.85
GO:CC	synapse	GO:0045202	3858_Glra2	57.14
GO:CC	cell junction	GO:0030054	3858_Glra2	53.72
GO:CC	neuron projection	GO:0043005	3858_Glra2	41.44
GO:MF	protein binding	GO:0005515	3858_Glra2	19.51
GO:MF	gated channel activity	GO:0022836	3858_Glra2	13.58
GO:MF	monoatomic ion channel activity	GO:0005216	3858_Glra2	12.19
HP	Epileptic encephalopathy	HP:0200134	3858_Glra2	1.66
KEGG	Axon guidance	KEGG:04360	3858_Glra2	8.92
KEGG	Oxytocin signaling pathway	KEGG:04921	3858_Glra2	6.14
KEGG	Aldosterone synthesis and secretion	KEGG:04925	3858_Glra2	5.31
REAC	Neuronal System	REAC:R-MMU-112316	3858_Glra2	17.64
REAC	Transmission across Chemical Synapses	REAC:R-MMU-112315	3858_Glra2	7.65
REAC	Axon guidance	REAC:R-MMU-422475	3858_Glra2	4.81
TF	Factor: CTCF; motif: NAGGGGGCGCNNKNNNN; match class: 1	TF:M08995_1	3858_Glra2	15.79
TF	Factor: ZF5; motif: NRNGNGCGCGCWN; match class: 1	TF:M00333_1	3858_Glra2	15.69
TF	Factor: FOXN4; motif: NNWANNCGWMCGCGTCNNNNMT; match class: 1	TF:M04662_1	3858_Glra2	15.56
WP	Splicing factor NOVA regulated synaptic proteins	WP:WP1983	3858_Glra2	5.19
WP	Calcium regulation in cardiac cells	WP:WP553	3858_Glra2	3.40
WP	Myometrial relaxation and contraction pathways	WP:WP385	3858_Glra2	2.58

Figure S8. Gene Ontology (GO) enrichment analysis for vulnerable dopaminergic population, Related to Figure4.

bioRxiv preprint doi: https://doi.org/10.1101/2024.12.20.629776; this version posted December 21, 2024. The copyright holder for this preprint (which was not certified by peer review) is the author/funder, who has granted bioRxiv a license to display the preprint in perpetuity. It is made available under aCC-BY 4.0 International license.

Enrichment analysis of gene ontology (GO) terms for key genes in a representative vulnerable dopamine cluster (A, 3854_Grin2c; B, 3855_Arhgap28; C, 3857_Anxa1; D, 3858_Glra2).

bioRxiv preprint doi: https://doi.org/10.1101/2024.12.20.629776; this version posted December 21, 2024. The copyright holder for this preprint (which was not certified by peer review) is the author/funder, who has granted bioRxiv a license to display the preprint in perpetuity. It is made available under aCC-BY 4.0 International license.

Figure S9.

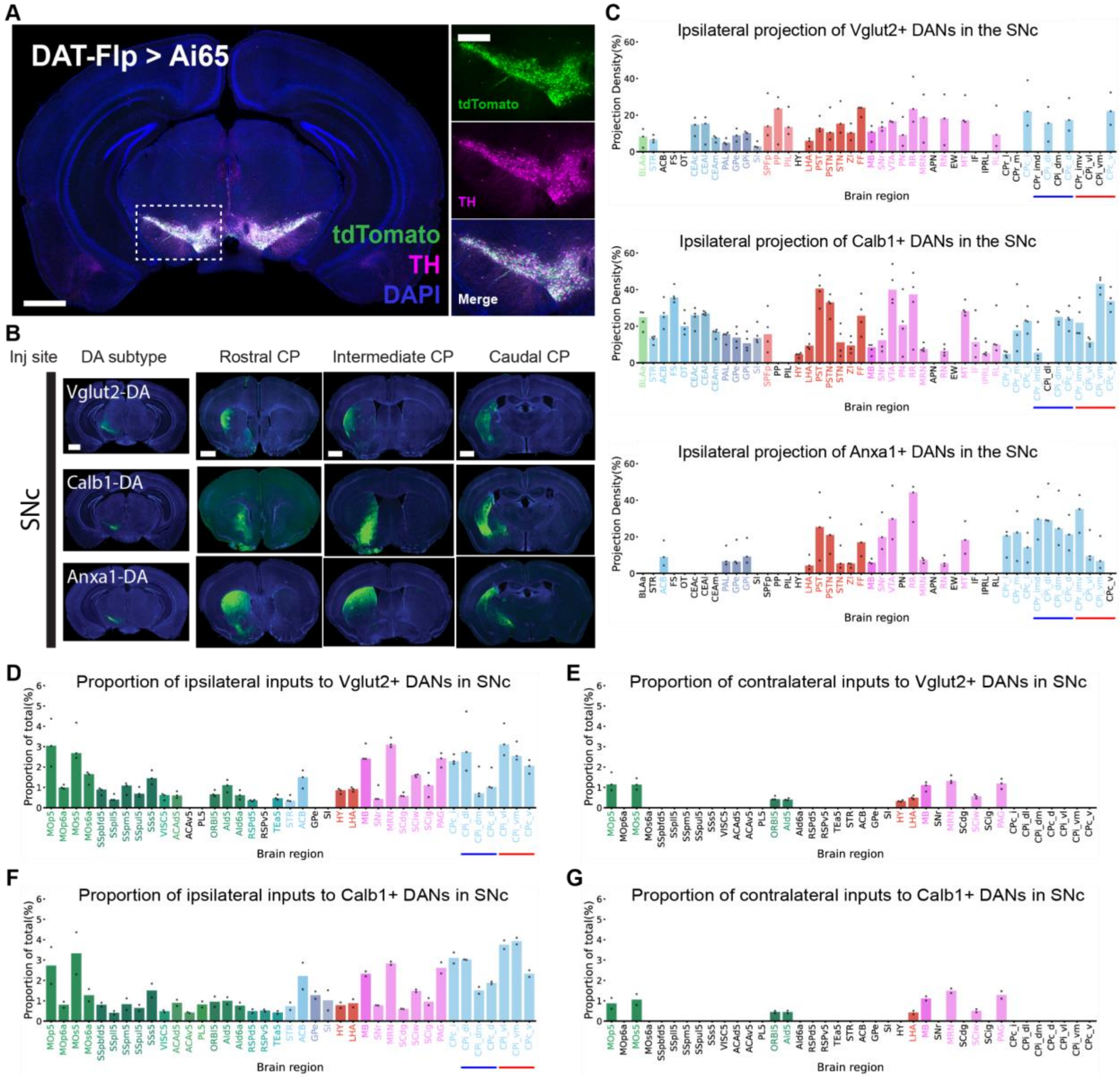

Figure S9. Whole-brain efferent and afferent connectivity of dopaminergic neuron subtypes, Related to Figure5.

A. Characterization of the DAT-Flp line using a reporter line (Ai65) confirmed that tdTomato+ cells are *Th*+ neurons. Scale bars: 1mm for the striatum (left) and 400μm for the substantia nigra (right). **B.** Representative images of axonal projections of *Vglut2*+, *Calb1*+ and *Anxa1*+ dopamine neuron subtypes in the SNc. They preferentially project to dorsolateral striatum, ventral medial striatum and dorsal striatum. Scale bar 1mm. **C.** Brain regions to which *Vglut2*+, *Calb1*+ and *Anxa1*+ dopamine neuron project, measured as the fraction of neurites found within those brain structures defined Allen brain atlas (n=4 *Calb1*+ and n=3 for *Vglut2*+ and *Anxa1*+).

bioRxiv preprint doi: https://doi.org/10.1101/2024.12.20.629776; this version posted December 21, 2024. The copyright holder for this preprint (which was not certified by peer review) is the author/funder, who has granted bioRxiv a license to display the preprint in perpetuity. It is made available under aCC-BY 4.0 International license.

Blue horizontal line indicates dorsal striatum and red line indicates ventral striatum. **D-G.** Statistical analysis of the whole-brain distribution of ipsilateral (left) or contralateral (right) monosynaptic inputs to *Vglut2*+ neurons (D, E) or *Calb1*+ dopamine neuron subtype (F, G) in the SNc. Brain areas are color-coded by the Allen Brain Atlas.

bioRxiv preprint doi: https://doi.org/10.1101/2024.12.20.629776; this version posted December 21, 2024. The copyright holder for this preprint (which was not certified by peer review) is the author/funder, who has granted bioRxiv a license to display the preprint in perpetuity. It is made available under aCC-BY 4.0 International license.

Figure S10.

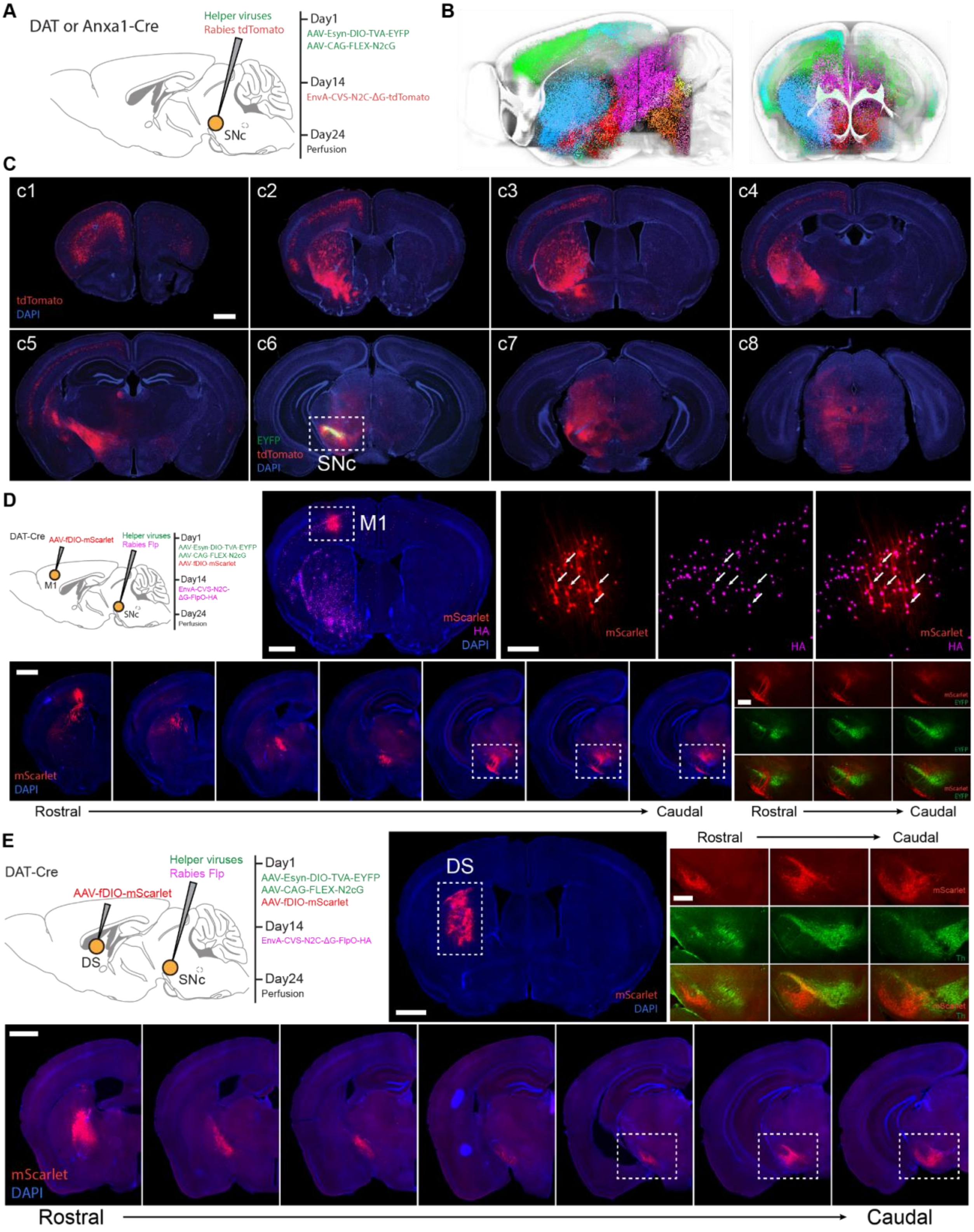

Figure S10. Direct afferent inputs from the motor cortex or striatum to the SNc DANs, Related to Figure5.

bioRxiv preprint doi: https://doi.org/10.1101/2024.12.20.629776; this version posted December 21, 2024. The copyright holder for this preprint (which was not certified by peer review) is the author/funder, who has granted bioRxiv a license to display the preprint in perpetuity. It is made available under aCC-BY 4.0 International license.

A. Schematic of the rabies injection procedure and experimental timeline for helper viruses and rabies viruses in the DAT-Cre or Anxa1-Cre. **B.** Whole-brain 3D reconstruction using BrainJ, color coded by brain regions (ref, left: sagittal view, right: coronal view). **C.** Representative images of monosynaptic inputs to DANs from the whole brain (c1-c8, from the rostral to caudal). The small white rectangle indicates the injection site, the SNc. Scale bar 1mm. **D-E.** Monosynaptic projections from primary inputs include cells associated with motor function to the SNc DANs (D, motor cortex; E, striatum). Scale bars: 1mm for the striatum and 400μm for the substantia nigra.
